# Cloning and expression of the *pkg1* gene from the GH55 family of the mycoparasite *Pestalotiopsis kenyana* PG52

**DOI:** 10.3389/fmicb.2025.1665330

**Published:** 2025-10-23

**Authors:** Mengling Yan, Wenjing Sui, Chen Chen, Ruotian Gao, Jinqiu Li, Jing Li

**Affiliations:** College of Biology and Food Engineering, Southwest Forestry University, Kunming, Yunnan, China

**Keywords:** *Pestalotiopsis kenyana*, β-1,3-glucanase, *pkg1*, bioinformatics, biological activity

## Abstract

**Introduction:**

β-1,3-glucanases are involved in degrading the cell wall of phytopathogenic fungi and can be used to control plant diseases. Our research group previously predicted that *Pestalotiopsis kenyana* PG52 has more glycoside hydrolase 55 (GH55) family genes than *Pestalotiopsis* sp. CR013. Therefore, their identification and expression were analyzed to screen the glucanase genes that may be involved in mycoparasitism.

**Methods:**

Using bioinformatics methods, the GH55 gene family was identified and predicted in the PG52 strain. According to the expression level of the gene induced by aeciospores, the GH55 family gene *pkg1*, which may be involved in mycoparasitism, was screened for cloning and expression. The expressed protein was purified, and its activity and ability to destroy aeciospores were determined.

**Results:**

There were seven GH55 family genes from the PG52 genome. An endo-β-1,3-glucanase gene, *pkg1*, which may have a mycoparasitic effect, was identified. The pkg1 gene was 2,304 bp long and expressed a stable 784 amino acid (aa) extracellular protein in *Escherichia coli Rosetta* (DE3). The enzyme activity of PKG1 was 4.88 U/mL, with laminarin as the substrate. The optimum temperature for PKG1 was approximately 60 °C, while the highest activity was at pH 7.0 ~ 9.0, and it exhibited destructive activity against aeciospore walls.

**Discussion:**

The β-1,3-glucanase gene from *P. kenyana* was successfully cloned and showed activity against aeciospores, which highlights its probable role in the mycoparasitic activity of *P. kenyana*, suggesting a new source of enzymes for biological control strategies that target fungal cell walls.

## 1 Introduction

Plant rusts are fungal diseases caused by rust fungi that are widely distributed and cause severe damage, affecting not only the ornamental value of garden plants but also causing significant crop yield reductions and seriously harming the agricultural economy (Huang L. et al., 2022). Due to its complex infection process and the widespread dispersal of aeciospores (Zhao et al., 2021), rust disease is very difficult to control. Although chemical agents can effectively treat rust diseases, they may cause resistance in pathogenic bacteria, contaminate the environment, and threaten biosecurity. Therefore, microorganisms with bacteriostatic ability have become the focus of biological control (Oliver, 2014; Cook et al., 2021). Studies have shown that seven secondary metabolites from *Aspergillus candidus* and *Aspergillus montenegroi* crude extracts are more than 95% effective against wheat rust (Ngo et al., 2021). *Pestalotiopsis* spp. were isolated from different parts of plant rust (Li et al., 2017a,b); through the inoculation experiment, it was found that these *Pestalotiopsis* spp. could destroy aeciospores and have potential for biological control.

β-1,3-glucanase (EC 3.2.1.58), a class of enzymes that hydrolyze β-1,3-glucans, is one of the pathogenesis-related (PR) proteins (Sels et al., 2008; Liang et al., 2020). It enables plants to disrupt the cell wall of pathogenic fungi and, in synergy with ligninase and chitinase, significantly inhibits the growth of pathogenic fungi (Hassan, 2014; Zhang et al., 2019; Wang et al., 2021b). The known β-1, 3-glucanases belong to 12 glycoside hydrolase (GH) families, including GH16, GH17, GH55, GH64, GH81, GH128, GH132, and others (Gastebois et al., 2013; Jia et al., 2021). Glycoside hydrolase 55 (GH55) contains both endo- and exo-β-1,3-glucanases, and the founder structure of this family is *PcLam55A* from the white rot fungus *Phanerochaete chrysosporium* (Bianchetti et al., 2015). Exo-β-1,3-glucanase belonging to the GH55 family has been shown to play a comprehensive role in the conidial maturation of *A. fumigatus* (Millet et al., 2019). AcGluA is a member of the GH55 family of endoglucanases, which significantly inhibits *Magnaporthe oryzae* blast at high doses (Wang et al., 2021b).

Current research and applications have focused on changes in β-1,3 glucanase activity during plant infestation (Liu et al., 2010; Serfling et al., 2016; Liang et al., 2020; Salcedo-Sarmiento et al., 2021). In addition to plants, microorganisms, such as *A. fumigatus* and *Trichoderma asperellum*, can also use β-1,3-glucanase as an antifungal agent (Hartl et al., 2011; Da Silva Aires et al., 2012). Therefore, mycoparasitic fungi that produce β-1,3-glucanase have attracted much attention, and studies on fungi, especially those with repressive ability, have focused on *Trichoderma* spp. (Qin et al., 2008).

*Pestalotiopsis* species occur commonly as plant pathogens and endophytes (Wang et al., 2015; Chen et al., 2021; Qi et al., 2021). In previous studies, we found that *Pestalotiopsis kenyana* PG52 and *Pestalotiopsis* sp. CR013 exhibit mycoparasitism against plant rusts (Gao et al., 2021). The PG52 strain, isolated from leaves of rust-infected heather, was found to inhibit common phytopathogenic fungi (Li et al., 2017b). Moreover, the mode of action of the PG52 strain is different from that of *Trichoderma* spp. in terms of mycoparasitism. Presumably, it is mainly toxin action (Xie et al., 2015), but the role of cell wall-degrading enzymes should not be neglected (Zhang et al., 2021). Zhang et al. (2021) analyzed the carbohydrase in the genome of the PG52 strain and found that the number of β-1,3-glucanase genes was much higher than that in *Trichoderma* spp. There are a few reported studies on the cloning and expression of the GH55 family of β-1,3-glucanase genes in *P. kenyana*.

This study identified seven GH55 family genes in the PG52 strain and successfully cloned *pkg1*, expressing an endoglucanase that is destructive to aeciospores. The mycoparasitic mechanism of the PG52 strain is discussed in this study, providing a theoretical foundation for the development of biopesticides in PG52.

## 2 Materials and methods

### 2.1 Microbial materials, plasmids, and growth conditions

The fungal strain *P. kenyana* PG52 (Accession Number: GCA_018092595.1) was isolated from aeciospores and preserved at Southwest Forestry University, Kunming, China (Sui et al., 2020). The Trizol Extraction kit, *Escherichia coli Rosetta* (DE3), pET28a(+) vector, anti-His mouse monoclonal antibody, and goat anti-rabbit IgG-HRP conjugate were purchased from Sangon, Shanghai, China. The pMD18-T vector was purchased from Takara, Kyoto, Japan. The PG52 strain was maintained on potato dextrose agar (PDA) at 25 °C for 7 days and inoculated into liquid-modified Fries medium (0.1% KH_2_PO_4_, 0.05% MgSO_4_·7H2O, 0.01% NaCl, 0.013% CaCl_2_·2H_2_O, 0.1% NH_4_NO_3_, 2% fructose, and 0.5% ammonium L-tartrate) (Li et al., 2017b). *E. coli Rosetta* (DE3) was grown in Luria–Bertani medium (1% peptone, 0.5% yeast extract, and 0.5% NaCl).

### 2.2 Identification, bioinformatics, and structure analysis of the glycoside hydrolase 55 family

Using known pfam12708 GH55 family gene sequences, the *P. kenyana* genome was searched with the BLASTp function of the NCBI. In addition, using the three databases of the dbCAN website, the *P. kenyana* genome genes were predicted, and the sequences annotated to the GH55 family were screened out. Two partial sequences were merged, and duplicates were deleted. Using the NCBI Conserved Domains Search^1^ and SMART,^2^ the screened sequences were verified by domain prediction. We used ProtParm^3^ to predict the basic physicochemical properties of the encoded protein online. Signal peptide sites were predicted using the SignalP software.^4^

### 2.3 Induction and expression analysis of the GH55 family genes

The PG52 strain was inoculated in liquid-modified Fries medium for 48 h at 28 °C and 130 rpm. Subsequently, 0.25% inactivated *Uromyces trifolii-repentis* aeciospores were added and cultured (Mei et al., 2022). Mycelia induced by rust spores at different time periods (0, 24, 48, and 72 h) (Tang et al., 2007) were collected and flash-frozen in liquid nitrogen, and then the mycelia were sent to Biomarker Technologies for transcriptome sequencing. Total RNA was extracted from the mycelia using Trizol reagent (Tiangen Biotech, Beijing, China) following the manufacturer’s protocol. Briefly, the frozen mycelia were ground into a powder in liquid nitrogen, homogenized with Trizol reagent, and subjected to phase separation using chloroform. The aqueous phase was collected, and RNA was precipitated with isopropanol, washed with 75% ethanol, and resuspended in RNase-free water. The purity, concentration, and integrity of the RNA sample were examined using NanoDrop, Qubit 2.0, and Agilent 2,100 systems. Qualified RNA samples were used to construct a cDNA library through several rounds of PCR. In this project, differentially expressed genes (DEGs) were identified using the criteria of a fold change of ≥2 and a *p*-value of < 0.01. A total of 12 samples were processed for transcriptome sequencing, generating 81.99 Gb of clean data. The qualified library was pooled based on pre-designed target data volume and then sequenced using the Illumina sequencing platform. The transcriptome raw data (Accession number: PRJNA951933) have been uploaded to the NCBI database.

After induction by aeciospores, the expression of the GH55 family genes was preliminarily analyzed with reference to the transcriptome results (Mei et al., 2022). The genes with high expression levels and significant expression changes were selected for real-time quantitative PCR (RT-qPCR). Total RNA was extracted from PG52 mycelium induced by aeciospores at different time points, and it was reverse transcribed to synthesize cDNA after verifying its integrity and purity. Using specific primers (Table 1) with a 10 μL reaction system (containing 5 μL of 2 × SYBR^®^ Green Supermix, 0.5 μL of each forward and reverse primer, 1 μL of cDNA, and 3 μL of ddH₂O), amplification on a PCR instrument was performed with pre-denaturation at 95 °C for 30 s, followed by 40 cycles (denaturation at 95 °C for 5 s, annealing at 58 °C for 30 s, and extension at 72 °C for 30 s), including three technical replicates and melting curve analysis. The relative expression level was calculated using the 2^−^ΔΔCT method, with Actin as the internal reference, and differences were analyzed using SPSS 26.0. The stable expression gene, Actin (XM_007836323.1), was used as an internal reference gene for the normalization of mRNA levels in this experiment; primer sequences are listed in Table 1, while the qPCR components and volumes are detailed in Table 2.

**Table 1:** RT-qPCR primer information.

Primers	5′ → 3’
*Actin*-F	AGATCATTGCTCCTCCTG
*Actin*-R	CACATTTGCTGGAAGGTC
*pkg1*-F	GATAACCTCCAGGCATTC
*pkg1*-R	CCATCCTCGGTCACTATA
*pkg3*-F	TTTGGTGGTGGTCTCTAC
*pkg3*-R	AGTCCTCAATGCTGACAA

**Table 2:** RT-qPCR components and volumes.

Components	Final concentration	Loading volume (μL)
2 × SYBR® Green Supermix	1×	5
Reverse primer	200 nM	0.5
Sense primer	200 nM	0.5
cDNA	N/A	1
ddH_2_O	N/A	3
Total		10

A total of three technical replicates were set up for each cell sample. Relative gene expression was automatically calculated using the qPCRsoft 3.2 software. The software applied the Pfaffl method (Pfaffl, 2001) and used the following formula:

$\text{Ratio}=\frac{{\left(1+E_{\text{target}}\right)}^{\mathrm{\Delta Ct}\text{target}(\text{Control}-\text{expt})}}{{1+E_{\mathrm{reference}})}^{\mathrm{\Delta Ct}\text{references}(\text{Control}-\text{expt})}}$

Relative mRNA levels were calculated using the comparative ΔCT value method: ΔCT = (CT target – CT Actin). Relative gene expression was determined using the 2^−ΔΔCT^ method.

### 2.4 Cloning of the β-1,3-glucanase gene

Total RNA from the mycelia of PG52 was extracted using the Trizol Extraction kit (Tiangen Biotech, Beijing, China), then RNA concentration was detected using NanoDrop 2000, and RNA quality was detected by 1.5% agarose gel electrophoresis. The total RNA was reverse transcribed to obtain cDNA using the M-MuLV First Strand cDNA Synthesis Kit (Sangon, Shanghai, China). PCR amplification was performed at 95 °C for 3 min, followed by 33 cycles (94 °C for 30 s, 58 °C for 30 s, 72 °C for 1.5 min, and extension for 7 min at 72 °C). The PCR amplification products were electrophoresed (1.0% agarose gel), purified, cloned into the pMD18-T vector, and sequenced by Sangon Biotech (Shanghai, China). The primers used for amplification were *RT985-1F*: GAATTCACAGACGGCCAGCAACAACAACAGC and *RT985-2247R*: CTCGAGTCAAGGTGTGTAGCGGCCGACGT.

### 2.5 Structural analysis, multiple sequence alignment, and phylogenetic analysis

Protein secondary structure was predicted using SOPMA^5^ (Geourjon and Deléage, 1995), and the 3D structure of the protein was predicted using I-TASSER^6^ (Zhou et al., 2022). The best model predicted was selected and visualized using PyMOL (Seeliger and De Groot, 2010). TBtools was used for drawing and beautification (Chen et al., 2020). To ensure sequence identity, the pkg. protein sequence was aligned with known GH55 family protein sequences found in other fungi using the DANMAN software. The phylogenetic tree of the GH55 family, as well as another phylogenetic tree of GH16, GH17, GH55, GH64, and GH81 families, was constructed using the MEGA 11.0 software. Evolutionary relationships were inferred using the neighbor joining method and a bootstrap of 1,000 replications, and evolutionary distances were computed using the Poisson correction method.

### 2.6 Expression, purification, and renaturation of the recombinant PKG1 protein

A recombinant PKG1 protein was generated by transforming the *E. coli Rosetta* (DE3) strain with pET28a-*pkg1* or pET28a (+) (empty vector control). The recombinant plasmid pET28a-*pkg1* was transformed into *E. coli Rosetta* (DE3) competent cells, which were then spread on a plate with kanamycin (30 μg/mL) after heat shock at 42 °C. Single colonies were randomly selected and inoculated in LB liquid medium supplemented with kanamycin (30 μg/mL). When the OD₆₀₀ reached 0.6, 0.5 mM IPTG was added to the LB medium, and the cultures were incubated at 15 °C overnight or 37 °C for 6 h. Uninduced cultures served as negative controls.

Cell samples were collected by 1,200 × *g* centrifugation for 10 min and sonication, and the cells were resuspended in 30 mL of cell lysis buffer and lysed by ultrasonication (power 140 W, 3 s on, 5 s off, total time 30 min, on ice). Meanwhile, the cell debris was precipitated by centrifugation (4,500 × *g* for 15 min at 4 °C), and the supernatant crude protein was collected for protein purification using Ni-column affinity chromatography. Protein purification was separated using SDS-PAGE (12%) gel and Coomassie brilliant blue R250 (1%) to detect the recombinant pET28a-*pkg1*. Immediately following this, the second protein was purified using a Q Sepharose High-Performance anion exchange column (GE Healthcare) and then analyzed using 12% SDS-PAGE and Western blot analysis. The primary and secondary antibodies were the anti-His mouse monoclonal antibody (Sangon, Shanghai, China) and the goat anti-rabbit IgG-HRP conjugate (Sangon, Shanghai, China). The recombinant PKG1 was renatured using different concentrations (8, 4, 2, and 1 mol/L for 6 h) of urea, following the renature conditions described by Shen et al. (2022).

### 2.7 Determination of enzyme activity and its effect on aeciospores

The enzyme activity analysis was performed using the 3,5-dinitrosalicylic acid (DNS) method, and 0.5% laminarin was used as the reaction substrate to determine the amount of reducing sugar hydrolyzed by PKG1 from the substrate. The experimental group contained 0.5 mL of the laminarin Tris-NaCl (50 mM) solution. We added 1 mL of the renatured crude enzyme solution. The mixture was incubated at 25 °C for 10 min; then, 1.5 mL of DNS was added and incubated in boiling water for 10 min (Huang X. et al., 2022). A total of three parallel experimental groups and one blank control group were set up, and 1 mL of the inactivated enzyme solution was added to the control group. A standard curve was established with glucose OD540, and enzymatic activity was calculated. One unit of enzyme activity (U/mL) refers to the amount of enzyme required to release the substrate to generate 1 μmol of reducing sugar per minute.

$X=\frac{C\times N}{V\times T\times 180}$ (Tang et al., 2007).

Here, X = enzyme activity (U/mL); C = amount of glucose (mL) obtained from the standard curve; N = sample dilution factor; V = amount of enzyme involved in the reaction (mL); and T = reaction time (minutes).

To determine the optimum reaction temperature, enzyme activity was measured at pH 5.0 across a temperature range of 20–80 °C (preheated for 10 min). The relative activity at each temperature was calculated by setting the maximum activity observed as 100%. For optimum pH determination, reactions were conducted at 25 °C using the 5% laminarin substrate prepared in buffers covering a pH range of 3.0–11.0 (citrate–phosphate buffer for pH 2.2–8.0; sodium carbonate–bicarbonate buffer for pH 9.0–10.0; and sodium carbonate-hydroxide buffer for pH 11.0). Relative activities were similarly calculated by normalizing to the maximum activity observed at the optimal pH.

Aeciospores were added to an 80 μg/mL enzyme solution to distribute them evenly (Tang et al., 2007). After 1, 2, 4, 6, and 8 days of treatment, a portion of the suspension was aspirated for filming. The aeciospores were soaked in 100 μL of sterile Tris–HCl buffer as a control and observed under a light microscope. The rust spores were stained with 0.4% trypan blue (Mei et al., 2022).

### 2.8 Statistical analysis

The data were analyzed using one-way ANOVA followed by the LSD multiple comparison test with the SPSS Statistics software (version 26.0) to compare the differences between the treatment and the control groups. Statistical significance was set at a *p*-value of <0.05 (^*^p < 0.05).

## 3 Results

### 3.1 Bioinformatics analysis of the GH55 family sequences

A total of seven GH55 family genes were identified from the PG52 genome (Table 3). The protein length varied from 766 amino acid (aa) residues (pkg4) to 1,426 aa (pkg2). Their molecular weights were 80.11–153.28 kDa. The pI values of the proteins encoded by *pkg1* to *pkg7* ranged from 4.04 to 6.14. Most of these proteins showed relatively acidic pI values, with five proteins (pkg1, pkg2, pkg3, pkg6, and pkg7) having pI values below 4.5. Only *pkg4* (5.11) and *pkg5* (6.14) exhibited slightly higher, yet still acidic to neutral-leaning pI values. All proteins were stable and extracellular. Four *pkg* genes contained signal peptide regions.

**Table 3:** Analysis of the GH55 family sequences.

Gene ID	Protein length (aa)	Molecular mass (Da)	pI	SignalP	Subcellular localization	Accession number
pkg1	767	80110.3	4.36	+	extr	ON009317
pkg2	1,426	153283.88	4.42	−	extr	OQ450379
pkg3	970	100393.47	4.27	+	extr	OQ450380
pkg4	766	80210.44	5.11	+	extr	OQ450381
pkg5	826	88779.46	6.14	+	extr	OQ450382
pkg6	1,293	140060.37	4.13	−	extr	OQ450383
pkg7	1,129	122302.46	4.04	−	extr	OQ450384

extr, extracellular.

**Figure 1:**
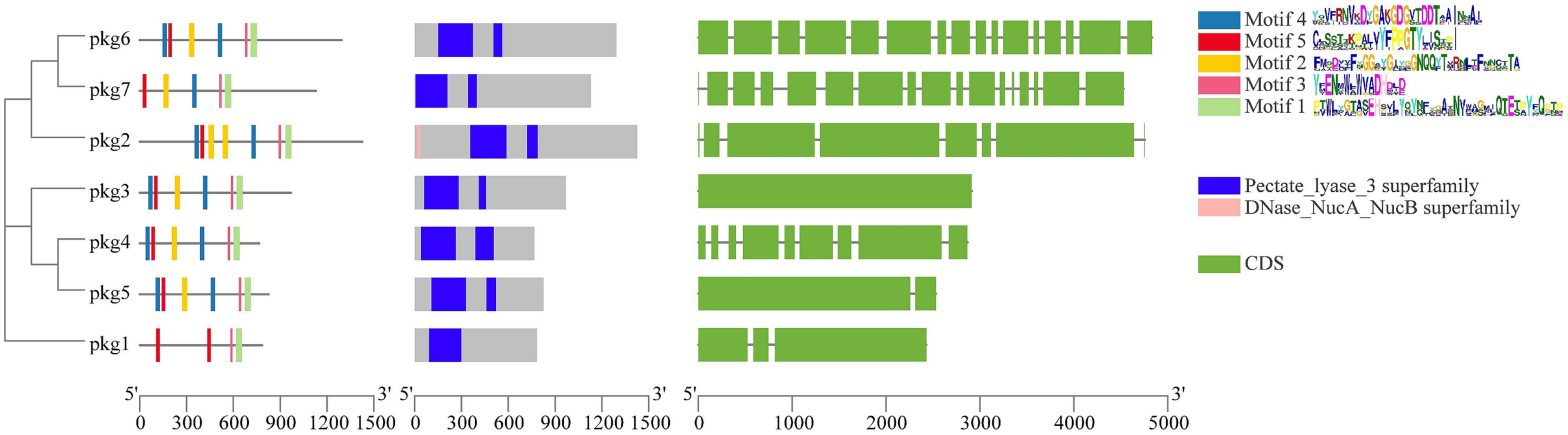
Phylogenetic tree, protein motifs, protein domains, and gene structure prediction of the GH55 family sequences from PG52.

The domains, motifs, and gene structures of the seven GH55 family sequences were predicted (Figure 1). The results showed that all sequences contained the Pectate_lyase_3 superfamily domain. In addition, only *pkg1* contained one conserved domain, and only *pkg2* had a DNase_NucA_NucB domain. All sequences contained Motif1, Motif3, and Motif5. Only *pkg1* did not contain Motif2 and Motif4. In the cluster analysis, *pkg1* was also a special sequence. Among the seven sequences, *pkg6* and *pkg7* contained 16 and 17 exons, respectively, while *pkg3* did not contain introns. The NCBI BLAST result showed that only *pkg1* in PG52 belonged to the endo-β-1,3-glucanase family.

### 3.2 Analysis of GH55 family gene expression

**Figure 2:**
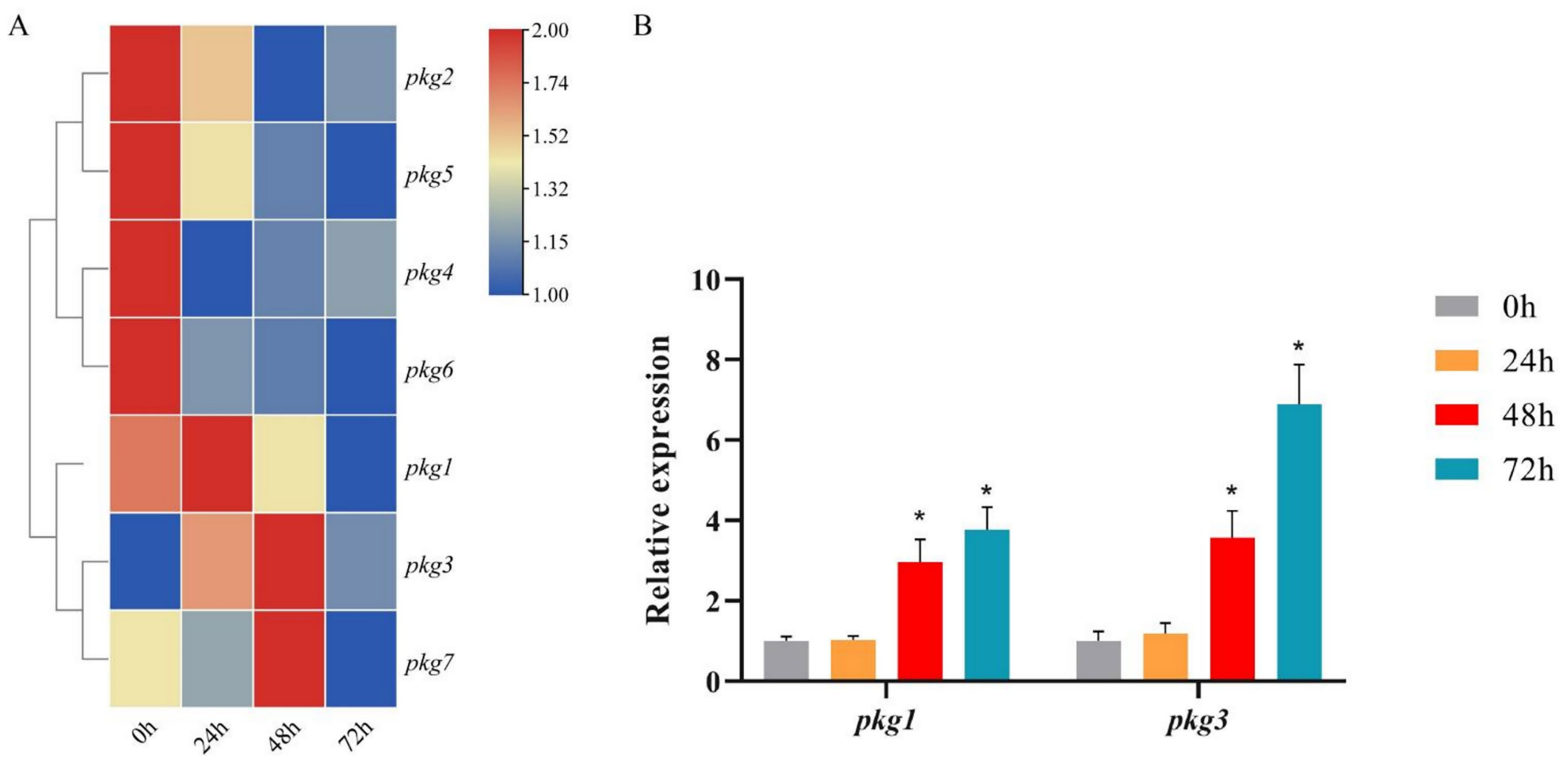
Expression pattern of the *pkg* genes under aeciospore induction. **(A)** Transcriptome FPKM results for the *pkg* genes under aeciospore induction, *n* = 3; **(B)** RT-qPCR results for *pkg1* and *pkg3*, *n* = 3, ^*^p < 0.05.

Upon induction with aeciospores, all seven *pkg* genes were expressed (Figure 2A). Most *pkg* genes exhibited downregulation within the first 24 h of induction. In contrast, *pkg1*, *pkg3*, and *pkg7* showed time-dependent upregulation, each peaking at distinct time points. Based on their high expression levels and significant fold changes, *pkg1* and *pkg3* were selected for RT-qPCR validation. The RT-qPCR results revealed dynamic expression patterns over time. During the initial 0–24 h post-induction, neither gene showed significant differential expression, with only minor fluctuations observed. However, at 48 h and 72 h post-induction, both genes were significantly upregulated, with *pkg3* exhibiting a > 7-fold increase at 72 h.

The RT-qPCR results differed from those of the transcriptome (Figure 2B). In RT-qPCR, the expression trends of *pkg1* and *pkg3* were consistent. Both genes were up-regulated at 48 h after induction, with expression levels peaking at 72 h, and there were significant differences. Based on the analysis of the seven *pkg* genes, we selected the gene that is both differentially expressed and the only endoglucanase-encoding gene for cloning experiments.

### 3.3 Cloning and bioinformatics analysis of *pkg1*

The 2,304 bp full-length open reading frame of *pkg1* was compared to the PG52 genome data using BLASTn, and the 100% matching degree confirmed that the *pkg1* gene was cloned successfully. *pkg1* encoded 767 amino acids with a calculated molecular mass of 82.0 kDa and pI = 4.55.

**Figure 3:**
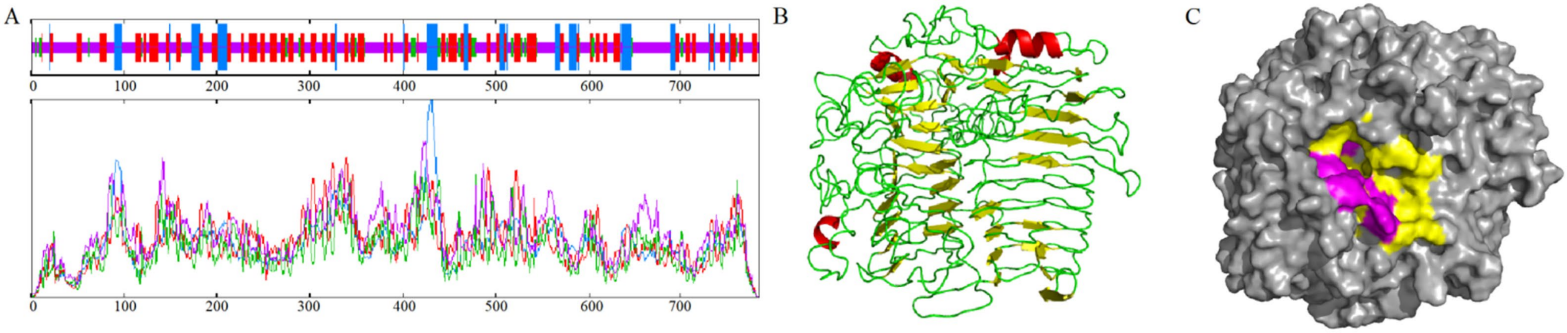
Sequence and structural analysis of PKG1. **(A)** Prediction of the PKG1 secondary structure sequence. **(B)** Prediction of the 3D structure of PKG1; yellow represents β-sheet, red represents *α*-helix, green represents loop. **(C)** Predicted substrate-binding cleft of PKG1; yellow represents the substrate-binding groove; purple represents the aromatic blocks.

The results showed that the PKG1 protein was predicted to be localized extracellularly and did not contain a transmembrane structure. Therefore, it is a non-transmembrane protein that does not migrate within cells. The proportions of PKG1 protein secondary structure components were as follows: alpha helix, 11.86%; extended strand, 31.76%; beta turn, 6.51%; and random coil, 49.87% (Figure 3A). The 3D structure of the PKG1 protein exhibited a (*β*/*α*)_8_ TIM barrel fold with structural similarity to Lam55A from *Phanerochaete chrysosporium* (Tao et al., 2013) and a β-(1,3)-glucanase from *Chaetomium thermophilum* (Papageorgiou et al., 2017), which includes two antiparallel beta sheets (Figure 3B). The predicted substrate-binding cleft of PKG1 is shown in Figure 3C. Purple represents the aromatic amino acid residues (Tyr 278, Typ 586, Typ 588, Tyr 651, Typ 714, Glu 716, Tyr 722, and Typ 775), where two amino acids (Typ 586 and Typ 588) close the substrate-binding cleft, ensuring that the substrate binds the protein in one direction.

### 3.4 Phylogeny relatedness of the pkgs protein sequences

**Figure 4:**
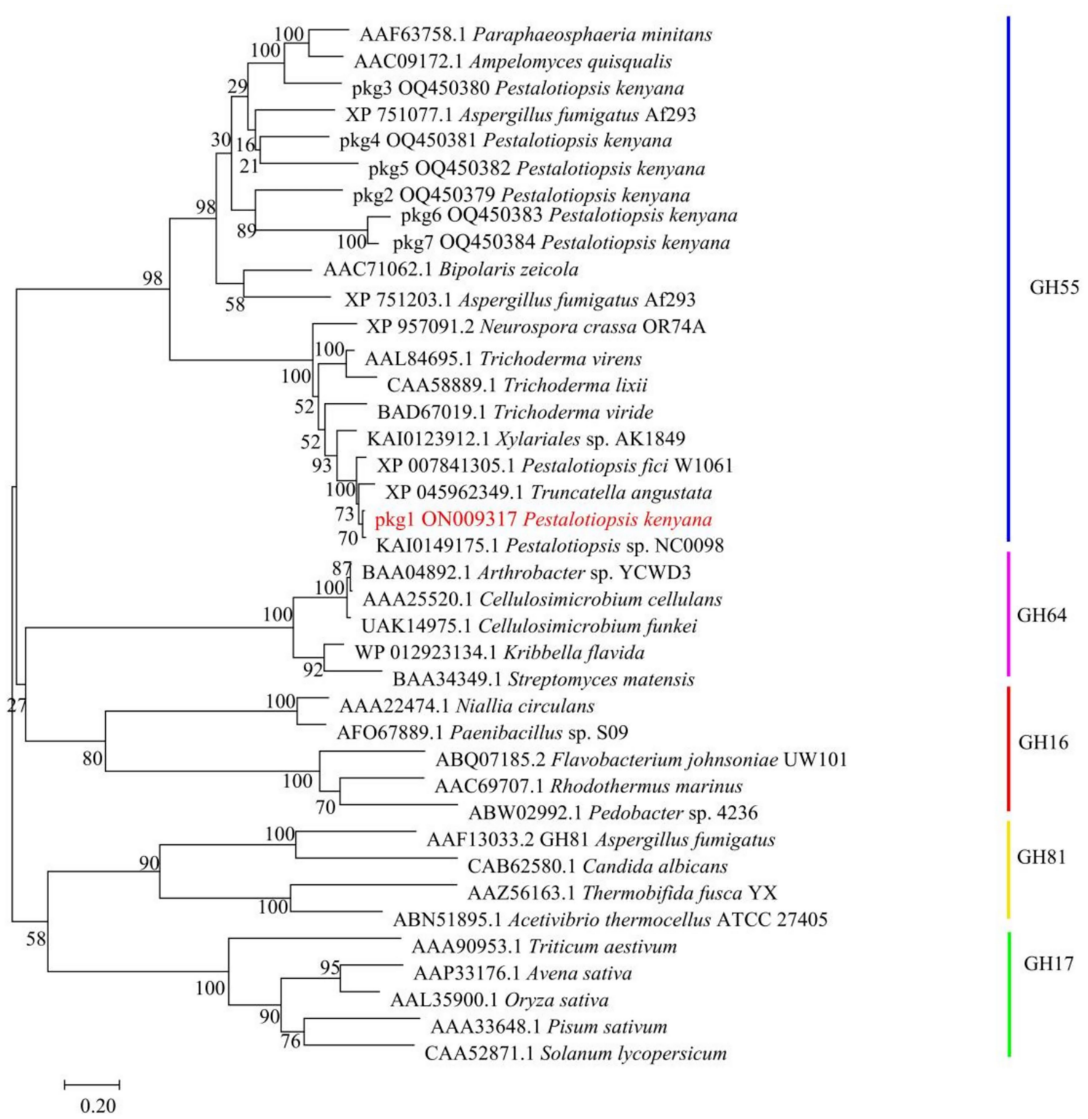
Clustering analysis of pkg. amino acid sequences with GH16, GH17, GH55, GH64, and GH81 family sequences. pkg1 is highlighted in red.

The seven GH55 family sequences were clustered with glucanase sequences from other known families, and the results showed that the candidate sequence pkg1 was located in the branch of the GH55 family (Figure 4). Among them, in the phylogenetic tree, the *Pestalotiopsis* sp. NC0098 glucanase sequence homology was highest. The cluster analysis of the amino acid sequences within the GH55 family showed that only pkg1 belonged to endo-β-1, 3-glucanase among the seven pkg. genes, and this is consistent with previous results.

### 3.5 Protein expression assays and purification

**Figure 5:**
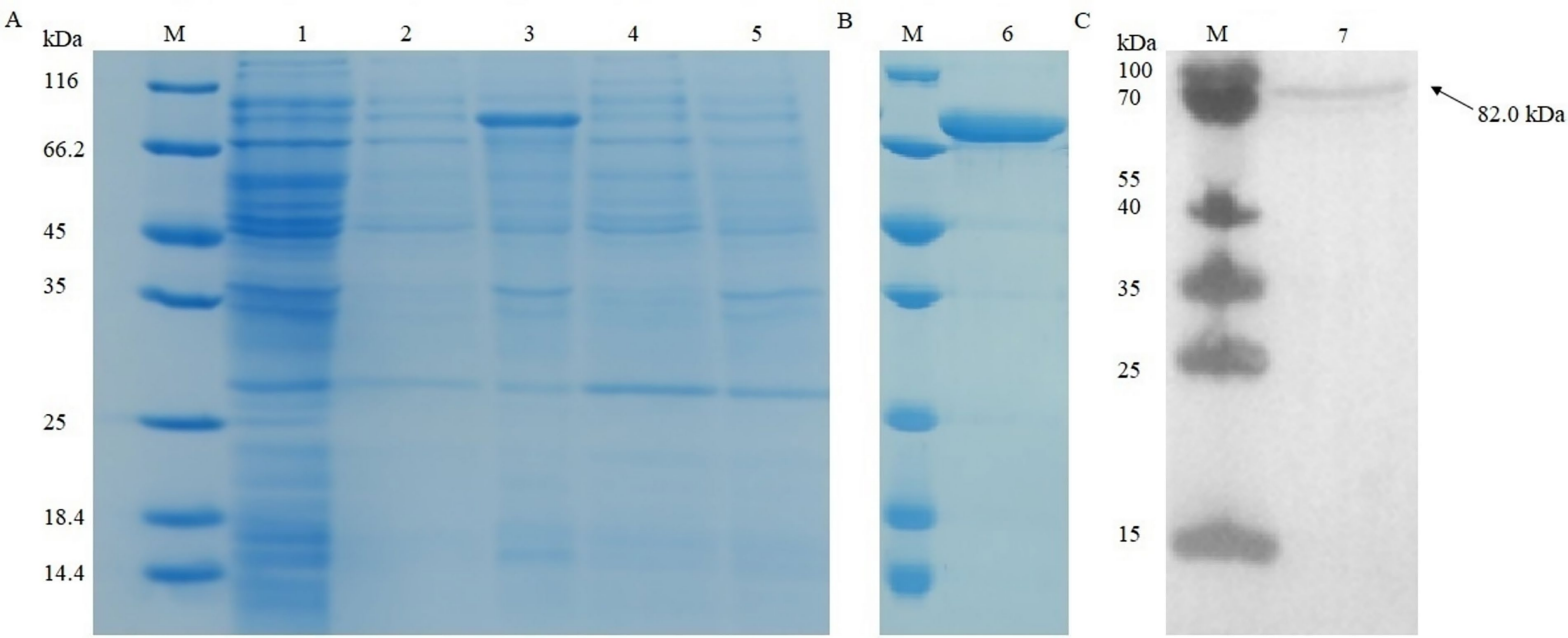
SDS-PAGE and Western blot analysis of recombinant PKG1. Recombinant protein expression analysis in **(A)**. Purified recombinant protein in **(B)**. Western blot analysis results of the supernatant induced at 15 °C in **(C)**. M: protein marker; 1: uninduced sample; 2: supernatant induced at 15 °C; 3: precipitation induced at 15 °C; 4: supernatant induced at 37 °C; 5: precipitation induced at 37 °C; 6: final purified protein; 7: supernatant induced at 15 °C.

The recombinant strains were cultured under different conditions (15 °C overnight and 37 °C for 6 h). After being ultrasonically disrupted, the supernatant and precipitate were collected for SDS-PAGE experiments (Figure 5A). The results showed that the optimal condition for *pkg1* expression in *E*. *coli* was 15 °C after overnight induction, and the protein was mainly distributed in the supernatant. The results showed that the protein with a molecular weight of approximately 82.0 kDa was successfully expressed, consistent with the expected results (Figure 5B). The resulting supernatant induced at 15 °C was used for Western blot analysis to examine recombinant PKG1 protein expression (Figure 5C).

### 3.6 Endo-glucanase activity, optimum temperature, and pH

**Figure 6:**
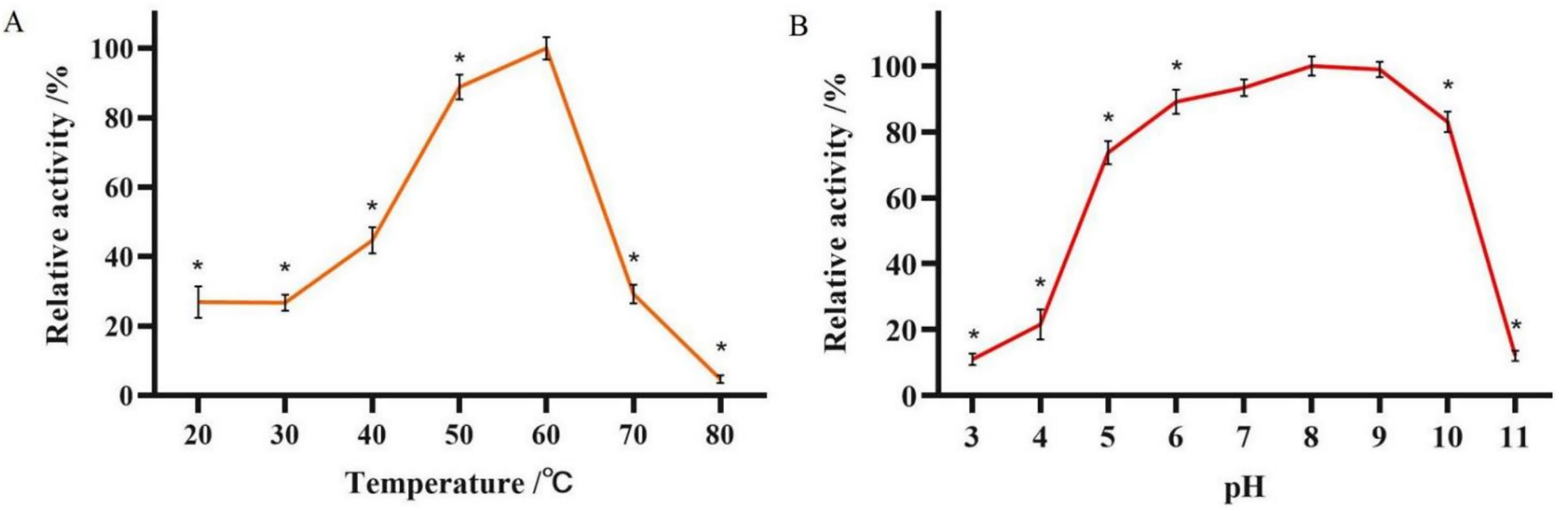
Determination of the optimum temperature and pH of the PKG1 protein. **(A)** Relative enzyme activity of PKG1 at various temperatures at pH 5.0. **(B)** The relative enzyme activity of PKG1 at different pH levels at 25 °C. *n* = 3, ^*^p < 0.05.

The enzymatic activity of glucanase PKG1 was 4.88 U/mL. As shown in Figure 6A, the optimal temperature screening experiment for the PKG1 protein showed that the enzyme activity of PKG1 was highest at 60 °C. Within the temperature range of 50 ~ 60 °C, the enzyme activity of PKG1 remained high. Below 50 °C, the enzyme activity was maintained at 20% ~ 45%. Above 60 °C, the enzyme activity decreased sharply, and the enzyme was inactivated at 80 °C. Therefore, the PKG1 protein is very sensitive to high temperature.

The results of the optimum pH measurement of PKG1 at 25 °C are shown in Figure 6B. The PKG1 protein showed no significant difference in activity within the pH range of 7 ~ 9. Within the pH range of 5 ~ 10, its relative enzyme activity remained above 70%, but the activity declined rapidly in other pH ranges, with only approximately 10% of relative activity observed. Figure 6B indicates that PKG1 showed good activity under a broad range of pH conditions, but its activity rapidly reduced under extreme pH conditions.

### 3.7 Enzyme activity against aeciospores

**Figure 7:**
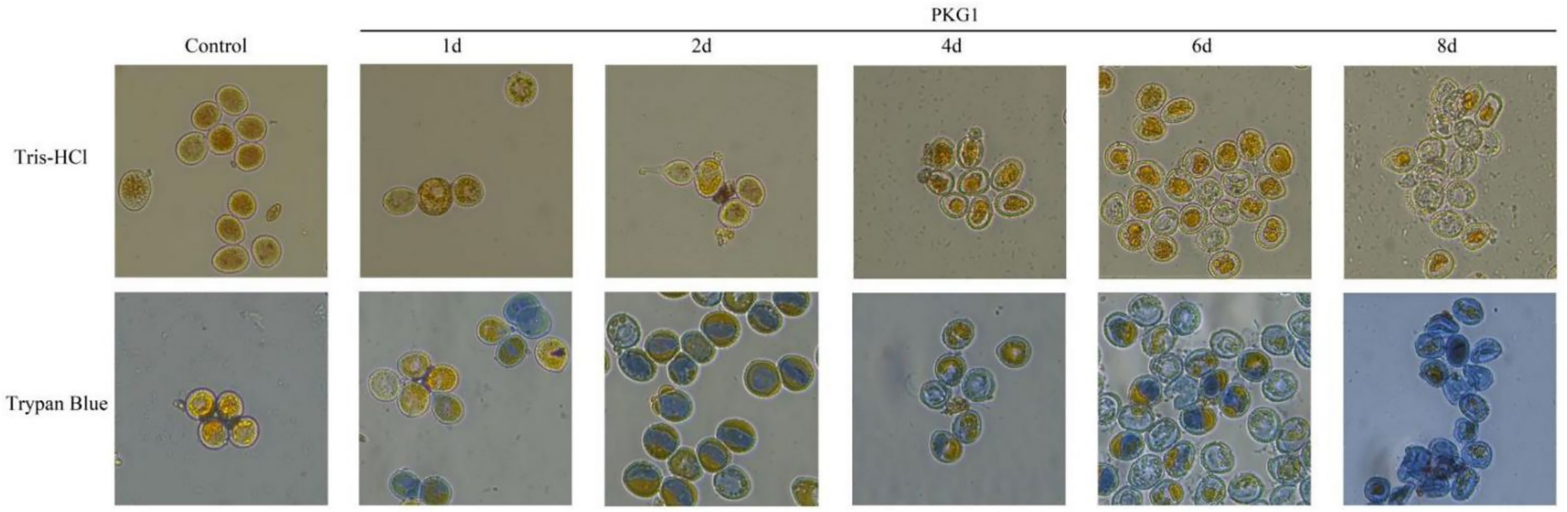
Destructive effects of PKG1 on the cell walls of aeciospores (25 °C). The living aeciospores and contents appear yellow, and the dead aeciospores are stained blue by trypan blue.

The enzyme activity assay result showed that the refolded PKG1 protein had β-1,3-glucanase and enzyme activity. The results of treating aeciospores at 25 °C are shown in Figure 7. In the control group, the rust spore wall was intact, the shape was round, and the contents were full and could not be stained by trypan blue. After being treated with the enzyme for 1 day, there was no significant change in the wall of the aeciospores, but after trypan blue staining, some aeciospores died. At 2 days of treatment, the aeciospore walls were complete, but the contents began to exude. After trypan blue staining, almost all aeciospores were dead, and the contents were in a squeezed state. At 4 days of treatment, in addition to the rupture of the aeciospore wall, the contents appeared to contract and either aggregate into granules or flow out of the cells, and the aeciospores were empty shells. At 6 days of treatment, the effect of the enzyme was very obvious, and most of the aeciospores had all the contents exuded, and only the empty shell remained. After staining, some of the aeciospores still contained contents separated from the cell wall but were dead. At 8 days of treatment, the proportion of the empty shell aeciospore wall increased, and all aeciospores could be stained. Therefore, the enzyme affected the aeciospores at 25 °C within 1 day, and the complete effect could be achieved at 6 days of treatment.

## 4 Discussion

Many glucanases from fungi exhibit broad antifungal abilities. For example, Ccglu17A, a functional exo-1,3-β-glucanase discovered from *Chaetomium cupreum* Ame, shows good antifungal activity and can inhibit the reproduction of pathogenic fungi (Jiang et al., 2017; Monteiro and Ulhoa, 2006). Purified β-glucanase from *Aspergillus niger* showed inhibitory effects on *Fusarium oxysporum* and *Penicillium digitatum* (El-Shora et al., 2021). The expression product of the first cloned endo-β-1,3-Glucanase *bgn13.1* gene from *Trichoderma harzianum* CECT 241 showed a hydrolytic effect on both yeast and filamentous fungal cell walls (De La Cruz et al., 1995; Marco and Felix, 2007). Burazerović et al. (2025) were the first to discover the hyperparasitic fungus *Arthrorhynchus nycteribiae* in Bosnia and Herzegovina and Montenegro, and they expanded its distribution range to Serbia. They revealed the spatiotemporal distribution pattern of this fungus in the bat fly *Penicillidia conspicua*, with the highest infection rate occurring in summer (23%). In addition, the infection rate in female bat flies was significantly higher than that in male bat flies (21% vs. 11%) (Burazerović et al., 2025). Tyagi et al. (2023) were the first to report that *Cladosporium oxysporum* is a potential new hyperparasitic fungus of the poplar rust fungus *Melampsora medusae*, which inhibits the germination of rust spores through enzymatic hydrolysis and direct penetration mechanisms, significantly reducing the infection rate. Li et al. (2025) reported that endophytic fungi (such as *Cladosporium*) form a mutualistic symbiotic relationship with plants by secreting effector proteins (such as β-1,3-glucanase) and altering the host’s immune response and that they may indirectly inhibit pathogenic fungi (Wang et al., 2021a). Zhao et al. (2021) found through transcriptome analysis that basidiospores and pycniospores of the wheat stripe rust fungus *Puccinia striiformis* specifically exhibit high expression of cell wall-degrading enzymes and mating-related genes, which may be related to their adaptability in infecting the alternate host Berberis (Zhan et al., 2023).

The results for the two genes were inconsistent between RNA-seq and RT-qPCR, and many studies also explained the plausibility of this situation (Kao et al., 2017; Huang and Zhou, 2022). The possible reasons for the discrepancies in our analysis are as follows: First, the difference in the estimation of changes in gene expression levels resulted from the different quantification methods of RT-qPCR and RNA-seq; however, this does not mean that the result of any method is wrong. Second, in the results of RNA-seq, the FPKM values of the seven GH55 family genes identified were low, with only *pkg1* and *pkg3* showing FPKM values above five. Low FPKM value may also affect the results of RT-qPCR and RNA-seq. Third, RNA-seq and RT-qPCR are two different experimental platforms. Due to different technical principles, it is reasonable that some results do not correspond exactly one-to-one. Everaert et al. (2017) research results showed that a difference of approximately 20% between the results of RNA-seq and RT-qPCR is reasonable.

Expressing the glucanase gene in *E. coli* may be problematic (Akcapinar et al., 2011). However, the prokaryotic expression system is a mature system and easy to cultivate, which can not only increase productivity but also reduce production costs (Tang et al., 2009). Zhang et al. (2022) used the pET-32a vector to help the disulfide bond fold correctly. Qin et al. (2008) showed that glycosylation is very important for the activity of glucanase. In this study, the enzymatically active PKG1 protein was successfully expressed in *E. coli*, indicating that post-translational modifications were possible but not necessary for PKG1. Similarly, studies have shown that the β-1,3-glucanase MoGluB from *Magnaporthe oryzae* can be highly expressed in *E. coli* and exhibit antifungal activity (Wang et al., 2021c). *T6-Echi18-5*, a chitinase expressed in *E. coli*, has also been identified to exhibit nematocidal activity (Shen et al., 2022). Although amino acid residues at the substrate binding site were predicted to be unmodified, the effect of protein modification on the PKG1 enzyme still needs to be verified by subsequent experiments.

PKG1 was found to have a wider range of pH adaptations at 25 °C, similar to CBM6E and Mzl86 (Lee et al., 2014; Jia et al., 2021), and exhibited its highest enzyme activity at higher temperatures, similar to rLamC27 (Zhou et al., 2019). According to Gao et al. (2021), the properties of some cloned and expressed glucanases were statistically analyzed, and the optimum temperature for most enzymes was below 50 °C. However, it was found that the optimal temperature of endoglucanase could reach 70 °C (Jaafar et al., 2020). The results of our study showed that the optimal temperature of the PKG1 enzyme was approximately 60 °C. When measuring enzyme activity *in vitro*, optimal pH results can vary due to the different properties of pH buffers and substrates. The MoGluB protein can maintain high relative activity across a broad pH range of 4 to 10. However, in PBS buffer, the enzyme activity of MoGluB was lower than in other acidic and alkaline buffers (Wang et al., 2021c). We can reasonably speculate that the buffers of different systems have an impact on enzyme activity. Since the PKG1 protein was obtained from a mycoparasite, we mainly used it to explore its role in biological control. Although PKG1 could not exert its maximum effect at the optimum temperature, the enzyme had good acid–base activity at 25 °C and could destroy aeciospores within 1 day at 25 °C. Further experiments should be conducted to verify whether the PKG1 protein has a broad inhibitory effect on plant pathogens and whether any other β-1,3-glucanase or related cell wall-degrading enzymes are involved in mycoparasitism. The concentration of 80 μg/mL PKG1 used in this study effectively degraded aeciospore walls within days. This concentration is comparable to, or even lower than, the effective doses reported for other antifungal glucanases and chitinases, which often range from 50 to 200 μg/mL (Li et al., 2022). This demonstrates the potent and efficient lytic activity of PKG1 against its target substrate. For future practical applications, the prospects are twofold: firstly, PKG1 could be developed into a novel enzymatic biocontrol agent. Achieving this will require optimizing large-scale production and formulating the enzyme to enhance its stability and persistence in the phyllosphere. Secondly, and perhaps more promisingly, the *pkg1* gene itself represents a valuable genetic resource. It could be engineered into plants to create transgenic crops with inherent, broad-spectrum resistance to rust and other fungal diseases by enhancing their ability to degrade pathogen cell walls.

Chitinases and glucanases can synergistically destroy the pathogenic fungi’s cell wall (Xue et al., 2021; Podgórska-Kryszczuk et al., 2022). Li et al. (2022) cloned and expressed CHI10, a chitinase gene from *Trichoderma atroviride*, and found that CHI10 can break up the walls of aeciospores. However, in this study, aeciospores treated with PKG1 hardly showed broken aeciospores. The rust spore walls were more of an empty shell state, which may be due to the different functions of chitin and glucan in the rust spore wall. There are also abundant GH18 family chitinase genes in the PG52 strain, which can be screened for cloning and expression to identify chitinases that can cooperate with the PKG1 protein, and their mechanism of action can be studied. Therefore, whether PKG1 can act synergistically with chitinase to destroy aeciospores is still unknown, and the PG52 chitinase needs to be analyzed in future studies.

In the previous study, we tested the PG52 strain to verify its mycoparasitic ability. The destructive effect of the PG52 strain on aeciospores emptied them; the contents were separated from the rust spore wall and concentrated into a mass (Li et al., 2017a; Sui et al., 2020). We also observed aeciospores parasitized by *Pestalotiopsis* sp. CR013 using scanning electron microscopy (Li et al., 2017b). Their inclusions were concentrated, and the cell wall was deformed but not broken. In addition, we isolated a toxin (3-nitropropionic acid) from PG52, which can change the membrane’s permeability and overflow the contents (Li et al., 2017a). The mechanism of PG52 mycoparasitism is a complex biological process resulting from the interaction of various enzymes and small molecules. According to the experimental results of this study, PKG1 played an important role in destroying host spores. To address the gap of *in vivo* validation critical for assessing PKG1’s practical utility, future research will focus on three key directions: first, conducting pot-scale greenhouse assays to evaluate PKG1’s rust-suppressive efficacy on susceptible crops (e.g., wheat, heather) via foliar spraying of formulated PKG1, with disease severity (pustule density) and crop growth quantified to confirm biocontrol efficiency; second, performing small-plot field trials in rust-endemic regions (e.g., Yunnan) to assess PKG1’s real-world performance, including leaf surface persistence and efficacy against natural rust populations, with optional synergy testing with PG52-derived chitinases; third, exploring stable *pkg1* expression in transgenic crops (e.g., wheat, poplar) to confer innate rust resistance, with greenhouse and field evaluations of resistance and yield stability. These steps will bridge the gap between current *in vitro* findings and PKG1’s practical agricultural application.

In summary, seven GH55 family genes were identified from the PG52 genome. According to the changes in expression levels, a glucanase gene *pkg1* was screened out and successfully cloned and expressed. After protein purification and inclusion body renaturation, we obtained an endo-β-1,3-glucanase with an enzyme activity of 4.88 U/mL, an optimum temperature of approximately 60 °C, and a pH of 5.0 ~ 10.0, which maintained 70% activity. pkg1 had a significant destructive effect on aeciospores and showed the potential to be prepared as a biological control enzyme agent, further highlighting the mechanism of mycoparasitism and providing a new scheme for plant disease control.

## Data Availability

Transcriptome raw data (Accession number: PRJNA951933) has been uploaded to the NCBI database.

## Footnotes

^1^ https://www.ncbi.nlm.nih.gov/Structure/cdd/wrpsb.cgi

^2^ https://smart.embl.de/

^3^ https://web.expasy.org/protparam/

^4^ https://services.healthtech.dtu.dk/service.php?SignalP-5.0

^5^ https://npsa-prabi.ibcp.fr/cgi-bin/npsa_automat.pl?page=npsa_sopma.html

^6^ https://zhanggroup.org/I-TASSER/
