# Supplementary material for: Cloning and expression of the pkg1 gene from the GH55 family of the mycoparasite Pestalotiopsis kenyana PG52
Source: Front Microbiol. 2025 Oct 23;16:1665330. doi: 10.3389/fmicb.2025.1665330 (PMC12590764; doi:10.3389/fmicb.2025.1665330)
Supplement: Supplementary file 1 [file Supplementary_file_1.docx]

**Basic Analysis Report of RNA-Seq with ParametersAbstract**

(1) A total of 12 were processed for transcriptome sequencing, generating 81.99Gb Clean Data. At least 6.35Gb clean data were generated for each sample with minimum 92.06% of clean data achieved quality score of Q30.

(2) Clean reads of each sample were mapped to specified reference genome. Mapping ratio ranged from 95.11% to 97.14%. Prediction of alternative splicing, gene structure optimization analysis and novel gene discovery was processed on top of mapping results, during which 2,397 were discovered and 101 novel genes were annotated with a putative function.

(3) In this project, differentially expressed genes (DEGs) were identified using the criteria of Fold Change≥2 and FDR<0.01. Then GO/KEGG enrichment, GSEA, differential alternative splicing (AS) and protein interactions of DEGs were analyzed. For more information please see below.

**1 Experimental Procedure**

As shown in the following figure, the workflow of mRNA sequencing includes sample preparation, library construction, library quality control and sequencing.

Fig1 mRNA sequencing workflow.

**1.1 RNA Quality Assessment**

Purity, concentration and integrity of RNA sample were examined by NanoDrop, Qubit 2.0, Agilent 2100, etc. Only RNA with good quality could move on to following procedures.

**1.2 Library Construction**

Qualified RNA were processed for library construction. The procedures are described as follow:

(1) mRNA was isolated by Oligo(dT)-attached magnetic beads.

(2) mRNA was then randomly fragmented in fragmentation buffer.

(3) First-strand cDNA was synthesized with fragmented mRNA as template and random hexamers as primers, followed by second-strand synthesis with addition of PCR buffer, dNTPs, RNase H and DNA polymerase I. Purification of cDNA was processed with AMPure XP beads.

(4) Double-strand cDNA was subjected to end repair. Adenosine was added to the end and ligated to adapters. AMPure XP beads were applied here to select fragments within size range of 300-400 bp.

(5) cDNA library was obtained by certain rounds of PCR on cDNA fragments generated from step 4.

**1.3 Library Quality Control**

In order to ensure the quality of library, Qubit 2.0 and Agilent 2100 were used to examine the concentration of cDNA and insert size. Q-PCR was processed to obtain a more accurate library concentration. Library with concentration larger than 2 nM is acceptable.

**1.4 Sequencing**

The qualified library was pooled based on pre-designed target data volume and then sequenced on Illumina sequencing platform.

**2 Analysis Flow**

Clean data with high quality was obtained by filtering Raw data, which removes adapter sequence and reads with low quality. These clean data were further mapped to pre-defined reference genome generating mapped data. Assessment on insert size and sequencing randomness were processed on mapped data as library quality control. Basic analysis on mapped data included gene expression quantification, alternative splicing analysis, novel genes prediction and genes structure optimization.

RNA sequencing bioinformatics pipeline was shown below:

Fig2 RNA sequencing bioinformatics pipeline

**3 Data Quality Control**

Based on sequencing-by-synthesis (Sequencing By Synthesis, SBS) technology, cDNA libraries were sequenced on Illumina high-throughput platform, generating significant amounts of high-quality data known as raw data. Raw data was saved in FASTQ format. Each sample has two FASTQ file, containing cDNA reads measured at both ends respectively.

Following figure shows a demo FASTQ file.

Fig3 Fastq format

Note: A FASTQ file normally contains four lines:
The first line begins with @ and is followed by sequence ID and an optional description.
The second line is a series of single letters representing sequence.
The third line begins with + and optional description.
The last line is the corresponding quality value of the bases in the second line. The length of this line should be exactly the same as Line 2. Base quality score is calculated as ASCII-33.

**3.1 Sequencing Quality Control**

It is crucial to ensure the quality of the reads before moving onto following analysis. Raw data contains useless data such as primers, adapters, etc., which need to be removed before analysis. Procedures for data quality control were listed as follow:

(1) Trim adapter contaminations

(2) Remove nucleotides with low Quality-score.

Data processed by above steps is named "Clean data". Clean data was provided in FASTQ format.

**3.2 Sequencing bases quality score**

Quality Score or Q-score represents the probability of an incorrect base. This Phred quality score is defined as following equation[[1]](file:///C:\Users\27846\Desktop\%E8%AE%BA%E6%96%87\%E9%9A%8B%E6%96%87%E9%9D%99\index_en.html#ref1):

*Q*=−10∗*log*10*P*

In the equation, P stands for the base calling error probabilities. Following table shows the relations between quality score and base calling accuracy：

Table1 Quality score and base calling accuracy

| **Phred Quality Score** | **Probability of incorrect base call** | **Base call accuracy** |
| --- | --- | --- |
| 10 | 1/10 | 90% |
| 20 | 1/100 | 99% |
| 30 | 1/1000 | 99.9% |
| 40 | 1/10000 | 99.99% |

Base call with higher Q-scores are believed to be more reliable and less likely to be error base. For example, Q20 is equivalent to the probability of one incorrect base call in 100 times.

Rate of error basing calling is influenced by the instrument, reagents, samples, etc. It is commonly found in Illumina platform that the rate slowly climbs along the reading of sequence due to the consumption of reagents. The high error rate at first six bases of reads are normally caused by inefficient binding between random hexamer primers and RNA templates.

Distribution of error rate along reads were shown in the following figures.

- A1.quality.png
- A2.quality.png
- A3.quality.png
- B1.quality.png
- B2.quality.png
- B3.quality.png
- C1.quality.png
- C2.quality.png
- C3.quality.png
- D1.quality.png
- D2.quality.png
- D3.quality.png

Fig4 Reads average error rate

Note: X-axis: Position on the reads. Y-axis: Average error rate on corresponding position.

Result directory:

[BMK_1_Reads_Control/BMK_1_Quality](file:///C:\\Users\\27846\\Desktop\\%E8%AE%BA%E6%96%87\\%E9%9A%8B%E6%96%87%E9%9D%99\\BMK_1_Reads_Control\\BMK_1_Quality)

**3.3 Nucleotide Distribution on Reads**

Nucleotide distribution test is designed to detect separation of AT and GC. In theory, according to the cDNA random fragmentation process and complementary base pairing principle, the frequency of A, T, G, C should be the same and steady along the reads. However, practically, fluctuations at 5'-end are commonly seen due to certain bias in binding of random haxemer primers and templates.

- A1.acgtn.png
- A2.acgtn.png
- A3.acgtn.png
- B1.acgtn.png
- B2.acgtn.png
- B3.acgtn.png
- C1.acgtn.png
- C2.acgtn.png
- C3.acgtn.png
- D1.acgtn.png
- D2.acgtn.png
- D3.acgtn.png

Fig5 Nucleotide distribution along reads

Note: X-axis: Position on reads. Y-axis: Percentage of certain nucleotide on corresponding position.

Result directory:

[BMK_1_Reads_Control/BMK_2_acgtn](file:///C:\\Users\\27846\\Desktop\\%E8%AE%BA%E6%96%87\\%E9%9A%8B%E6%96%87%E9%9D%99\\BMK_1_Reads_Control\\BMK_2_acgtn)

**3.4 Sequencing Data Statistics**

After quality control of sequencing data, 81.99Gb Clean Data were obtained and and more than 92.06% of bases in each sample had a Q-score no less than Q30.

Statistics of sequencing data was provided in the following table.

Table2 Sequencing data Statistics

| **Samples** | **Clean reads** | **Clean bases** | **GC Content** | **%≥Q30** |
| --- | --- | --- | --- | --- |
| A1 | 23,309,921 | 6,967,038,584 | 57.15% | 92.90% |
| A2 | 23,477,255 | 7,012,277,514 | 57.12% | 92.72% |
| A3 | 22,661,804 | 6,771,803,008 | 57.16% | 92.35% |
| B1 | 21,919,772 | 6,545,263,140 | 57.18% | 93.22% |
| B2 | 21,826,191 | 6,521,760,068 | 57.15% | 92.33% |
| B3 | 22,556,723 | 6,736,005,632 | 57.20% | 92.19% |
| C1 | 23,258,292 | 6,949,237,948 | 57.13% | 92.45% |
| C2 | 24,863,210 | 7,425,646,420 | 57.10% | 92.95% |
| C3 | 22,536,758 | 6,736,753,760 | 57.26% | 92.06% |
| D1 | 24,696,147 | 7,381,186,160 | 57.40% | 93.01% |
| D2 | 21,252,789 | 6,345,026,562 | 57.41% | 93.19% |
| D3 | 22,100,368 | 6,596,717,692 | 57.53% | 92.96% |

Note: (1)Samples: Sample name;
(2)Clean reads: Counts of clean PE reads;
(3)Clean bases: total base number of Clean Data;
(4)GC content: Percentage of G,C in clean data.
(5)≥Q30%: Percentage of bases with Q-score no less than Q30.

The relationship between the project sample name and the fq file name is shown in the following table:

Table3 Sample information table

| **#ID** | **read1_name** | **read2_name** |
| --- | --- | --- |
| A1 | Unknown_AY874-01T0001_good_1.fq | Unknown_AY874-01T0001_good_2.fq |
| A2 | Unknown_AY874-01T0002_good_1.fq | Unknown_AY874-01T0002_good_2.fq |
| A3 | Unknown_AY874-01T0003_good_1.fq | Unknown_AY874-01T0003_good_2.fq |
| B1 | Unknown_AY874-01T0004_good_1.fq | Unknown_AY874-01T0004_good_2.fq |
| B2 | Unknown_AY874-01T0005_good_1.fq | Unknown_AY874-01T0005_good_2.fq |
| B3 | Unknown_AY874-01T0006_good_1.fq | Unknown_AY874-01T0006_good_2.fq |
| C1 | Unknown_AY874-01T0007_good_1.fq | Unknown_AY874-01T0007_good_2.fq |
| C2 | Unknown_AY874-01T0008_good_1.fq | Unknown_AY874-01T0008_good_2.fq |
| C3 | Unknown_AY874-01T0009_good_1.fq | Unknown_AY874-01T0009_good_2.fq |
| D1 | Unknown_AY874-01T0010_good_1.fq | Unknown_AY874-01T0010_good_2.fq |
| D2 | Unknown_AY874-01T0011_good_1.fq | Unknown_AY874-01T0011_good_2.fq |
| D3 | Unknown_AY874-01T0012_good_1.fq | Unknown_AY874-01T0012_good_2.fq |

Note: #ID:sample name; read1_name、read2_name：Double-ended fq file name corresponding to the sample name. When the number of samples is large, the display may be incomplete, it is recommended to directly view the BMK_1_Reads_Control/BMK_3_Reads_stat/samples_name.xls file.

Result directory:

[BMK_1_Reads_Control/BMK_3_Reads_stat](file:///C:\\Users\\27846\\Desktop\\%E8%AE%BA%E6%96%87\\%E9%9A%8B%E6%96%87%E9%9D%99\\BMK_1_Reads_Control\\BMK_3_Reads_stat)

**4 Data Alignment to Reference Genome**

Reference genome was pre-defined for the analysis. Version information of the reference genome is: Pestalotiopsis_kenyana.customer_v1.0.genome.fa.

HISAT2[[2]](file:///C:\Users\27846\Desktop\%E8%AE%BA%E6%96%87\%E9%9A%8B%E6%96%87%E9%9D%99\index_en.html#ref2) is a highly efficient system for mapping RNA-seq reads, which is a more advanced version of TopHat2/Bowtie2.HISAT2 uses a Burrows-Wheeler Transform and Ferragina-Manzini（FM）index based search. HISAT2 uses one global graph FM index (GFM) to represent general population, as well as small indexes (local indexes) combined with several alignment strategies in order to achieve more efficient alignment.

StringTie[[3]](file:///C:\\Users\\27846\\Desktop\\%E8%AE%BA%E6%96%87\\%E9%9A%8B%E6%96%87%E9%9D%99\\index_en.html#ref3) was applied to assemble the mapped reads. The algorithm is established based on optimality theory. It utilizes a novel network flow algorithm as well as an optional de novo assembly step to assemble and quantify transcripts representing multiple spliced variants for each gene locus.

The workflow of analysis was shown in the figure below.

Fig6 Schematic flow of HISAT2

**4.1 Mapping Statistics**

Mapping ratio refers to the percentage of Mapped Reads in Clean Reads, which indicates the utilization of RNA data. Besides the influence of sequencing data quality, mapping ratio is also affected by the quality of reference genome assembly, biological classification relation between sequenced sample and reference subspecies. Mapping ratio is an important parameter to examine if reference genome is suitable for following bioinformatic analysis.

The mapping ratio of each sample against reference genome ranged from 95.11% to 97.14%.

Table4 Statistics on data mapping

| **Sample** | **Total Reads** | **Mapped Reads** | **Uniq Mapped Reads** | **Multiple Map Reads** | **Reads Map to '+'** | **Reads Map to '-'** |
| --- | --- | --- | --- | --- | --- | --- |
| A1 | 46,619,842 | 45,239,778 (97.04%) | 45,094,333 (96.73%) | 145,445 (0.31%) | 22,706,414 (48.71%) | 22,713,252 (48.72%) |
| A2 | 46,954,510 | 45,437,594 (96.77%) | 45,272,119 (96.42%) | 165,475 (0.35%) | 22,809,600 (48.58%) | 22,822,902 (48.61%) |
| A3 | 45,323,608 | 43,741,863 (96.51%) | 43,595,673 (96.19%) | 146,190 (0.32%) | 21,952,549 (48.44%) | 21,964,873 (48.46%) |
| B1 | 43,839,544 | 42,587,640 (97.14%) | 42,457,332 (96.85%) | 130,308 (0.30%) | 21,364,802 (48.73%) | 21,374,959 (48.76%) |
| B2 | 43,652,382 | 41,957,143 (96.12%) | 41,825,188 (95.81%) | 131,955 (0.30%) | 21,050,568 (48.22%) | 21,058,174 (48.24%) |
| B3 | 45,113,446 | 42,908,227 (95.11%) | 42,767,305 (94.80%) | 140,922 (0.31%) | 21,537,042 (47.74%) | 21,541,756 (47.75%) |
| C1 | 46,516,584 | 44,613,434 (95.91%) | 44,462,214 (95.58%) | 151,220 (0.33%) | 22,388,246 (48.13%) | 22,403,790 (48.16%) |
| C2 | 49,726,420 | 47,850,308 (96.23%) | 47,678,779 (95.88%) | 171,529 (0.34%) | 24,023,045 (48.31%) | 24,036,200 (48.34%) |
| C3 | 45,073,516 | 43,536,155 (96.59%) | 43,399,707 (96.29%) | 136,448 (0.30%) | 21,840,793 (48.46%) | 21,853,793 (48.48%) |
| D1 | 49,392,294 | 47,725,674 (96.63%) | 47,444,265 (96.06%) | 281,409 (0.57%) | 24,034,197 (48.66%) | 24,056,171 (48.70%) |
| D2 | 42,505,578 | 41,247,694 (97.04%) | 41,027,315 (96.52%) | 220,379 (0.52%) | 20,762,939 (48.85%) | 20,776,858 (48.88%) |
| D3 | 44,200,736 | 42,760,400 (96.74%) | 42,495,372 (96.14%) | 265,028 (0.60%) | 21,539,824 (48.73%) | 21,559,870 (48.78%) |

Note: Sample: sample ID in system;
Total Reads: Counts of Clean Reads, counted as single end;
Mapped Reads: Counts of mapped reads and the proportion of that in clean data;
Uniq Mapped Reads: Counts of reads mapped to a unique position on reference genome and proportion of that in clean data;
Multiple Mapped Reads: Counts of reads mapped to multiple positions on reference genome and proportion of that in clean data;
Reads Map to '+': Counts of reads mapped to the sense chain and the proportion of that in clean data;
Reads Map to '-': Counts of reads mapped to antisense chain and proportion of that in clean data.

Result directory:

[BMK_2_Genome_Alignment/BMK_1_Alignment_stat](file:///C:\\Users\\27846\\Desktop\\%E8%AE%BA%E6%96%87\\%E9%9A%8B%E6%96%87%E9%9D%99\\BMK_2_Genome_Alignment\\BMK_1_Alignment_stat)

**4.2 Summary on Mapping**

Distribution of coverage depth on reference genome was plotted based on the position of each mapped read on different chromosomes.

- A1.coverage.png
- A2.coverage.png
- A3.coverage.png
- B1.coverage.png
- B2.coverage.png
- B3.coverage.png
- C1.coverage.png
- C2.coverage.png
- C3.coverage.png
- D1.coverage.png
- D2.coverage.png
- D3.coverage.png

Fig7 Distribution of mapped reads on reference genome: position and depth

Note: X-axis: Position on chromosome; Y-axis: Log2 of coverage depth (coverage depth was defined as reads counted within a chromosome window of 10 kb in length); Blue represents + strand and green represents - strand.

By summarizing the number of reads mapped to different regions of genes on reference genome, i.e. exons, introns and intergenic regions. In theory, reads originated from mature mRNA should be aligned to exons. The ones mapped to introns may come from RNA precursor or intron retention(alternative splicing events). The ones aligned to intergenic regions may due to imperfect annotation of genome. the distribution pie chart of mapped reads on different gene regions were generated, which was shown below.

- A1.type.png
- A2.type.png
- A3.type.png
- B1.type.png
- B2.type.png
- B3.type.png
- C1.type.png
- C2.type.png
- C3.type.png
- D1.type.png
- D2.type.png
- D3.type.png

Fig8 Distribution of reads on different gene regions

Note: Genome was divided into exon, intron and intergenic regions, which are coloured differently. The size of each area indicates the proportion of that in total mapped reads.

Result directory:

[BMK_2_Genome_Alignment/BMK_2_PNG](file:///C:\\Users\\27846\\Desktop\\%E8%AE%BA%E6%96%87\\%E9%9A%8B%E6%96%87%E9%9D%99\\BMK_2_Genome_Alignment\\BMK_2_PNG)

**5 Library Quality Control**

It is crucial to assure a library of good quality in order to obtain a better output of RNA sequencing. To ensure the quality of library, following three quality examinations were performed on RNA library.

(1)Fragment randomness and degradation of RNA sample was estimated by checking the distribution of mapped reads on genome

(2)Length dispersion was examined by the length distribution of inserts

(3)Sufficiency of library Volume (or mapped reads) was examined by generating saturation curve between sampled mapped reads against genes identified within certain expression accuracy.

**5.1 mRNA Fragmentation Randomness Check**

Ideally, we expect the reads generated by sequencing to cover mRNA evenly, which strongly counts on a higher randomness mRNA fragmentation. The randomness mRNA fragmentation is largely guaranteed by a sufficient amount of sample, proper method and time on fragmentation,etc.

The randomness of mRNA fragmentation is examined by the distribution of mapped reads on each mRNA. The bases on the mRNA won't be read during sequencing if the mRNA is heavily degraded, i.e. there will be no reads mapped to the region. Therefore, the degradation of RNA sample can also be checked by distribution of mapped reads on transcripts. Following figure shown the distribution of mapped reads on transcripts.

- A1.randcheck.png
- A2.randcheck.png
- A3.randcheck.png
- B1.randcheck.png
- B2.randcheck.png
- B3.randcheck.png
- C1.randcheck.png
- C2.randcheck.png
- C3.randcheck.png
- D1.randcheck.png
- D2.randcheck.png
- D3.randcheck.png

Fig9 Distribution of mapped reads on mRNA

Note: X-axis: Normalized mRNA position; Y-axis: Percentage of reads mapped to corresponding region in total mapped reads. Since the length of mRNAs differs from each other, all mRNAs were divided into 100 parts in order to count the mapped reads in each part. The figure shows the sum of the percentage on all mRNAs.

Result directory:

[BMK_3_Library_Assessment/BMK_1_Randcheck](file:///C:\\Users\\27846\\Desktop\\%E8%AE%BA%E6%96%87\\%E9%9A%8B%E6%96%87%E9%9D%99\\BMK_3_Library_Assessment\\BMK_1_Randcheck)

**5.2 Length Distribution of Inserts**

The dispersion of insert length is an important parameter representing the quality of library construction, especially in purification by magnetic beads. The size of inserts were counted as the distance between the start and end point on reference genome in paired-end reads mapping.

In majority of eukaryotic genome, the DNA coding region is not continuous, i.e. the exons are divided by introns. However, in RNA sequencing, mature mRNA without introns is sequenced. In this case, when the reads cover the region cross introns, the distance between start and end point of reads on reference genome will be larger than the insert size. Therefore, these reads may form several small peaks in the distribution curve.

Insert length distribution of each sample was shown in the figures below.

- A1.insertSize.png
- A2.insertSize.png
- A3.insertSize.png
- B1.insertSize.png
- B2.insertSize.png
- B3.insertSize.png
- C1.insertSize.png
- C2.insertSize.png
- C3.insertSize.png
- D1.insertSize.png
- D2.insertSize.png
- D3.insertSize.png

Fig10 Distribution of insert length

Note: X-axis: Insert size (bp), which stands for the distance between the start and end point on reference genome in paired-end reads mapping, ranging from 0 to 800 bp. Y-axis: Number of inserts.

Result directory:

[BMK_3_Library_Assessment/BMK_2_InsertSize](file:///C:\\Users\\27846\\Desktop\\%E8%AE%BA%E6%96%87\\%E9%9A%8B%E6%96%87%E9%9D%99\\BMK_3_Library_Assessment\\BMK_2_InsertSize)

**5.3 Saturation Test on RNA Sequencing Data**

In order to ensure the sufficiency of data volume, saturation in gene recognition against data volume needs to be checked. Since there are only limited number of genes in a species as well as limited transcripts at certain point, the number of genes recognized will gradually reach saturation along with the increase of data size. Genes with higher expression are more likely to be identified and quantified. Therefore, larger data volume is required to quantify low abundant genes.

The saturation of mapped data on genes at different expression level can be mimicked by checking the increase in number of genes identified with the increase in mapped data size. The saturation curve was shown below.

- A1.Saturation.png
- A2.Saturation.png
- A3.Saturation.png
- B1.Saturation.png
- B2.Saturation.png
- B3.Saturation.png
- C1.Saturation.png
- C2.Saturation.png
- C3.Saturation.png
- D1.Saturation.png
- D2.Saturation.png
- D3.Saturation.png

Fig11 Saturation test on RNA sequencing data

Note: Mapped reads were divided into 10 fractions. By counting the number of genes identified at different expression level along with the increase of reads, the saturation curve was generated. X-axis: Percentage of sampled reads in total mapped reads; Y-axis: Percentage of genes identified in total genes at different expression level with error within 15% fpkm. The lines closer to 1.0 on Y-axis indicates a more saturated situation. Lines with different colours represents genes with different expression level.

Result directory:

[BMK_3_Library_Assessment/BMK_3_Saturation](file:///C:\\Users\\27846\\Desktop\\%E8%AE%BA%E6%96%87\\%E9%9A%8B%E6%96%87%E9%9D%99\\BMK_3_Library_Assessment\\BMK_3_Saturation)

**6 Alternative Splicing Prediction**

In gene expression processes, particular exons on pre-mRNA may be included in or excluded from the final, resulting in different versions of mature mRNA. In this case, multiple proteins with different structures and biological functions can be translated from these alternatively spliced mRNA originated from the same gene. The process described here is named alternative splicing.

StringTie[[3]](file:///C:\\Users\\27846\\Desktop\\%E8%AE%BA%E6%96%87\\%E9%9A%8B%E6%96%87%E9%9D%99\\index_en.html#ref3) was applied to assemble the mapped reads generated by Hisat2. ASprofile[[5]](file:///C:\\Users\\27846\\Desktop\\%E8%AE%BA%E6%96%87\\%E9%9A%8B%E6%96%87%E9%9D%99\\index_en.html#ref5) was employed to predict alternative splicing events in each samples and sorting them into 12 types. Typical alternative splicing scheme were shown in the figure below.

Fig12 Typical Alternative Splicing event

Note: (A) Skipped exon and Multi-exon SKIP; (B) Intron retention and Multi-Intron retention; (C) Alternative exon; (D) transcription start site; (E) transcription terminal site, the red was the type of alternative splicing event.

Alternative splicing events were classified into 12 types in ASProfile tool which were shown below:

(1) TSS: Alternative 5' first exon (transcription start site) the first exon splicing;

(2) TTS: Alternative 3' last exon (transcription terminal site) the last exon splicing;

(3) SKIP: Skipped exon(SKIP_ON,SKIP_OFF pair) single exon skipping;

(4) XSKIP: Approximate SKIP (XSKIP_ON,XSKIP_OFF pair) single exon skipping (fuzzy boundary);

(5) MSKIP: Multi-exon SKIP (MSKIP_ON,MSKIP_OFF pair) multi-exon skipping;

(6) XMSKIP: Approximate MSKIP (XMSKIP_ON,XMSKIP_OFF pair) multi-exon skipping (fuzzy boundary);

(7) IR: Intron retention (IR_ON, IR_OFF pair) single intron retention;

(8) XIR: Approximate IR (XIR_ON,XIR_OFF pair) single intron retention (fuzzy boundary);

(9) MIR: Multi-IR (MIR_ON, MIR_OFF pair) multi-intron retention;

(10) XMIR: Approximate MIR (XMIR_ON, XMIR_OFF pair) multi-intron retention (fuzzy boundary);

(11)AE: Alternative exon ends (5', 3', or both);

(12) XAE: Approximate AE variable 5' or 3' end (fuzzy boundary);

**6.1 Statistics of Alternative Splicing Events**

Statistics of predicted alternative splicing events were shown in the figures below.

- As_event_stat1_6.png
- As_event_stat7_12.png

Fig13 Statistics of alternative splicing types

Note: X-axis: Number of transcripts in specific alternative splicing type; Y-axis: 12 alternative splicing types.

Result directory:

[BMK_4_Alternative_Splicing/BMK_1_Number_stats](file:///C:\\Users\\27846\\Desktop\\%E8%AE%BA%E6%96%87\\%E9%9A%8B%E6%96%87%E9%9D%99\\BMK_4_Alternative_Splicing\\BMK_1_Number_stats)

**6.2 Alternative Splicing Pattern**

Table5 List of alternative splicing (AS) pattern

| **event_id** | **event_type** | **gene_id** | **chrom** | **event_start** | **event_end** | **event_pattern** | **strand** |
| --- | --- | --- | --- | --- | --- | --- | --- |
| 1,000,001 | TSS | gene-evm.model.tig00058927_pilon_pilon_pilon.1 | tig00058927_pilon_pilon_pilon | 3,033 | 3,278 | 3,278 | + |
| 1,000,002 | TTS | gene-evm.model.tig00058927_pilon_pilon_pilon.1 | tig00058927_pilon_pilon_pilon | 3,677 | 4,048 | 3,677 | + |
| 1,000,003 | TSS | gene-evm.model.tig00058927_pilon_pilon_pilon.10 | tig00058927_pilon_pilon_pilon | 33,566 | 33,853 | 33,853 | + |
| 1,000,004 | TTS | gene-evm.model.tig00058927_pilon_pilon_pilon.10 | tig00058927_pilon_pilon_pilon | 35,568 | 36,164 | 35,568 | + |
| 1,000,005 | TSS | gene-evm.model.tig00058927_pilon_pilon_pilon.11 | tig00058927_pilon_pilon_pilon | 36,750 | 37,123 | 37,123 | + |
| 1,000,006 | TTS | gene-evm.model.tig00058927_pilon_pilon_pilon.11 | tig00058927_pilon_pilon_pilon | 37,925 | 38,594 | 37,925 | + |
| 1,000,007 | TSS | gene-evm.model.tig00058927_pilon_pilon_pilon.13 | tig00058927_pilon_pilon_pilon | 41,995 | 42,185 | 42,185 | + |
| 1,000,008 | TTS | gene-evm.model.tig00058927_pilon_pilon_pilon.13 | tig00058927_pilon_pilon_pilon | 42,785 | 43,316 | 42,785 | + |
| 1,000,009 | TSS | gene-evm.model.tig00058927_pilon_pilon_pilon.14 | tig00058927_pilon_pilon_pilon | 58,915 | 59,014 | 58,915 | - |

Note: event_id: ID for AS;
event_type: Type of AS;
gene_id: Gene ID;
；
(4)symbol: Gene symbol；
chrom: Chromosome ID;
event_start: Starting position of AS;
event_end: Ending position of AS;
event_pattern: Pattern of AS;
strand: +/- strand.

Result directory:

[BMK_4_Alternative_Splicing/BMK_2_Structure_stats](file:///C:\\Users\\27846\\Desktop\\%E8%AE%BA%E6%96%87\\%E9%9A%8B%E6%96%87%E9%9D%99\\BMK_4_Alternative_Splicing\\BMK_2_Structure_stats)

**7 Gene Structure Optimization**

The accuracy of gene annotation gained from reference genome could be limited by the software, quality of data, etc. Therefore, it is necessary to process gene structure optimization on annotated genes. During this process, if continuous mapped reads were found outside the boundaries of original genes, the boundary of a gene may be corrected by extending untranslate region (UTR) to upper and down stream. In this project, 1,760 genes were optimized, which were listed in the following table.

Table6 Gene structure optimization

| **#GeneID** | **Locus** | **Strand** | **Site** | **OriginalRegion** | **OptimizeRegion** |
| --- | --- | --- | --- | --- | --- |
| gene-evm.model.tig00000005_pilon_pilon_pilon.1 | tig00000005_pilon_pilon_pilon:2559-3816 | - | 5' | 3551-3551 | 3551-3816 |
| gene-evm.model.tig00000005_pilon_pilon_pilon.1002 | tig00000005_pilon_pilon_pilon:3071911-3073653 | + | 3' | 3072699-3072699 | 3072699-3073653 |
| gene-evm.model.tig00000005_pilon_pilon_pilon.1014 | tig00000005_pilon_pilon_pilon:3104694-3109671 | + | 3' | 3109505-3109505 | 3109505-3109671 |
| gene-evm.model.tig00000005_pilon_pilon_pilon.1049 | tig00000005_pilon_pilon_pilon:3209905-3212959 | + | 3' | 3211593-3211593 | 3211593-3212959 |
| gene-evm.model.tig00000005_pilon_pilon_pilon.105 | tig00000005_pilon_pilon_pilon:278058-282315 | + | 3' | 282305-282305 | 282305-282315 |
| gene-evm.model.tig00000005_pilon_pilon_pilon.1050 | tig00000005_pilon_pilon_pilon:3215674-3224717 | - | 3' | 3216110-3216110 | 3215674-3216110 |
| gene-evm.model.tig00000005_pilon_pilon_pilon.1060 | tig00000005_pilon_pilon_pilon:3237813-3239497 | + | 3' | 3239450-3239450 | 3239450-3239497 |
| gene-evm.model.tig00000005_pilon_pilon_pilon.1061 | tig00000005_pilon_pilon_pilon:3246973-3250429 | + | 3' | 3248805-3248805 | 3248805-3250429 |
| gene-evm.model.tig00000005_pilon_pilon_pilon.1077 | tig00000005_pilon_pilon_pilon:3300864-3306721 | - | 3' | 3301205-3301205 | 3300864-3301205 |

Note: GeneID: Gene ID;
Locus: Gene locus (chromosome ID: starting position-ending position);
Strand: +/- strand;
Site: Site of optimization (on 3' UTR or 5'UTR); OriginalRegion: Starting and ending position of original annotated genes;
OptimizedRegion: Starting and ending position of optimized genes.

Result directory:

[BMK_5_Gene_Structure_Optimize](file:///C:\\Users\\27846\\Desktop\\%E8%AE%BA%E6%96%87\\%E9%9A%8B%E6%96%87%E9%9D%99\\BMK_5_Gene_Structure_Optimize)

**8 Novel Gene Analysis**

**8.1 Novel Gene Discovery**

In order to optimize the annotation information of a genome, discovery of novel transcripts and genes was achieved by StringTie on base of reference genome. The mapped reads were assembled and compared with original annotations of the genome. The transcript regions without annotation obtained by above processes are defined as novel transcripts. Excluding short transcripts(coding peptides with less than 50 amino acids) or those containing only one exons, 2,397 novel genes were discovered in this project. GFF file of novel genes was shown below.

Table7 GFF file of novel genes

| **#Seq_ID** | **Source** | **Type** | **Start** | **End** | **Score** | **Strand** | **Phase** | **Attributes** |
| --- | --- | --- | --- | --- | --- | --- | --- | --- |
| tig00000005_pilon_pilon_pilon | StringTie | gene | 274 | 2,352 | . | + | . | ID=Pestalotiopsis_kenyana_newGene_1 |
| tig00000005_pilon_pilon_pilon | StringTie | mRNA | 362 | 2,352 | . | + | . | ID=Pestalotiopsis_kenyana_newGene_1.2;Parent=Pestalotiopsis_kenyana_newGene_1 |
| tig00000005_pilon_pilon_pilon | StringTie | exon | 362 | 1,294 | . | + | . | Parent=Pestalotiopsis_kenyana_newGene_1.2 |
| tig00000005_pilon_pilon_pilon | StringTie | exon | 1,353 | 2,352 | . | + | . | Parent=Pestalotiopsis_kenyana_newGene_1.2 |
| tig00000005_pilon_pilon_pilon | StringTie | mRNA | 423 | 2,352 | . | + | . | ID=Pestalotiopsis_kenyana_newGene_1.3;Parent=Pestalotiopsis_kenyana_newGene_1 |
| tig00000005_pilon_pilon_pilon | StringTie | exon | 423 | 475 | . | + | . | Parent=Pestalotiopsis_kenyana_newGene_1.3 |
| tig00000005_pilon_pilon_pilon | StringTie | exon | 617 | 2,352 | . | + | . | Parent=Pestalotiopsis_kenyana_newGene_1.3 |
| tig00000023_pilon_pilon_pilon | StringTie | gene | 2,200,211 | 2,200,807 | . | + | . | ID=Pestalotiopsis_kenyana_newGene_10 |
| tig00000023_pilon_pilon_pilon | StringTie | mRNA | 2,200,211 | 2,200,807 | . | + | . | ID=Pestalotiopsis_kenyana_newGene_10.1;Parent=Pestalotiopsis_kenyana_newGene_10 |

Note: #Seq_ID: Chromosome ID;
Source: Source of annotation (normally StringTie);
Type: Annotation Feature;
Start/End: Starting and ending position of the feature;
Score: Confidence of the annotation ("." represents a null value);
Strand: +/- strand of the feature;
Phase: phase of CDS feature (only available for CDS); be either "0", "1" or "2"; "." indicates not available;
Attributes: All the other information pertaining to this feature.

Besides supplementary information in genome annotation, FASTA file of novel gene sequences were provided, as shown in the following documents.

Result directory:

[BMK_6_New_Gene/BMK_1_NewGene_prediction](file:///C:\\Users\\27846\\Desktop\\%E8%AE%BA%E6%96%87\\%E9%9A%8B%E6%96%87%E9%9D%99\\BMK_6_New_Gene\\BMK_1_NewGene_prediction)

**8.2 Functional Annotation of Novel Genes**

Novel genes were annotated by DIAMOND[[8]](file:///C:\Users\27846\Desktop\%E8%AE%BA%E6%96%87\%E9%9A%8B%E6%96%87%E9%9D%99\index_en.html#ref8) against databases including NR[[9]](file:///C:\Users\27846\Desktop\%E8%AE%BA%E6%96%87\%E9%9A%8B%E6%96%87%E9%9D%99\index_en.html#ref9), Swiss-Prot[[10]](file:///C:\Users\27846\Desktop\%E8%AE%BA%E6%96%87\%E9%9A%8B%E6%96%87%E9%9D%99\index_en.html#ref10), COG[[11]](file:///C:\Users\27846\Desktop\%E8%AE%BA%E6%96%87\%E9%9A%8B%E6%96%87%E9%9D%99\index_en.html#ref11), KOG[[12]](file:///C:\Users\27846\Desktop\%E8%AE%BA%E6%96%87\%E9%9A%8B%E6%96%87%E9%9D%99\index_en.html#ref12) and KEGG[[13]](file:///C:\Users\27846\Desktop\%E8%AE%BA%E6%96%87\%E9%9A%8B%E6%96%87%E9%9D%99\index_en.html#ref13). KEGG Orthology of novel genes were obtained by above processes. GO[[14]](file:///C:\Users\27846\Desktop\%E8%AE%BA%E6%96%87\%E9%9A%8B%E6%96%87%E9%9D%99\index_en.html#ref14) Orthology of novel genes were obtained by the underlying software InterProScan[[15]](file:///C:\\Users\\27846\\Desktop\\%E8%AE%BA%E6%96%87\\%E9%9A%8B%E6%96%87%E9%9D%99\\index_en.html#ref15) basic on the InterPro database. The amino acid sequences of novel genes were blasted against Pfam[[16]](file:///C:\\Users\\27846\\Desktop\\%E8%AE%BA%E6%96%87\\%E9%9A%8B%E6%96%87%E9%9D%99\\index_en.html#ref16) database by HMMER[[17]](file:///C:\Users\27846\Desktop\%E8%AE%BA%E6%96%87\%E9%9A%8B%E6%96%87%E9%9D%99\index_en.html#ref17) to gain the annotation information.

Summary of annotated novel genes by each database were shown in the table below.

Table8 Summary of annotated novel genes

| **Annotated databases** | **New Gene Number** |
| --- | --- |
| COG | 4 |
| GO | 54 |
| KEGG | 22 |
| KOG | 5 |
| Pfam | 29 |
| Swiss-Prot | 11 |
| TrEMBL | 93 |
| eggNOG | 45 |
| nr | 99 |
| All | 101 |

Note: Annotated databases: Database applied; New Gene Number: Number of annotated genes in specific database.

Result directory:

[BMK_6_New_Gene/BMK_2_NewGene_anno](file:///C:\\Users\\27846\\Desktop\\%E8%AE%BA%E6%96%87\\%E9%9A%8B%E6%96%87%E9%9D%99\\BMK_6_New_Gene\\BMK_2_NewGene_anno)

**9 Gene Expression Quantification**

**9.1 Gene Expression Quantification**

The number of fragments from a transcript is affected by sequencing data volume (or number of mapped reads), length of the transcript, expression level of transcripts. In order to reveal the expression level of each transcript more accurately, the number of mapped reads needs to be normalized by the length of its transcripts. FPKM(Fragments Per Kilobase of transcript per Million fragments mapped) was applied to measure the expression level of a gene or transcript by StringTie using maximum flow algorithm. The equation for FPKM is shown below.

*FPKM*=*cDNAFragmentsMappedFragments*(*Millions*)∗*TranscriptLength*(*kb*)

In the equation, cDNA Fragments represents the number of PE reads mapped to the specific transcript; Mapped Fragments (Millions) is the number of all mapped reads, which is counted as 10^6; Transcript Length(kb) is the length of transcript in unit of 10^3 b.

Table9 Quantification of gene expression

| **#ID** | **A1** | **A2** | **A3** | **B1** | **B2** | **B3** | **C1** | **C2** | **C3** | **D1** | **D2** | **D3** |
| --- | --- | --- | --- | --- | --- | --- | --- | --- | --- | --- | --- | --- |
| gene-evm.model.tig00000005_pilon_pilon_pilon.623 | 139.49 | 156.91 | 149.51 | 84.37 | 104.55 | 89.19 | 111.61 | 107.85 | 149.22 | 134.34 | 125.51 | 122.27 |
| gene-evm.model.tig00000005_pilon_pilon_pilon.622 | 95.08 | 98.10 | 95.14 | 140.44 | 138.15 | 129.73 | 114.44 | 121.40 | 109.60 | 84.68 | 96.89 | 86.93 |
| gene-evm.model.tig00000005_pilon_pilon_pilon.621 | 78.89 | 75.38 | 80.69 | 110.48 | 103.74 | 112.29 | 84.39 | 75.99 | 80.68 | 39.54 | 32.90 | 33.76 |
| gene-evm.model.tig00000005_pilon_pilon_pilon.620 | 29.31 | 28.25 | 28.31 | 34.43 | 35.84 | 35.58 | 36.40 | 36.21 | 30.87 | 22.15 | 26.40 | 21.63 |
| gene-evm.model.tig00000005_pilon_pilon_pilon.627 | 1.01 | 1.24 | 0.89 | 0.58 | 0.45 | 0.51 | 0.61 | 1.18 | 0.41 | 0.78 | 1.11 | 0.65 |
| gene-evm.model.tig00000005_pilon_pilon_pilon.626 | 4038.23 | 3972.98 | 3816.43 | 3555.37 | 3968.19 | 2867.58 | 3928.74 | 3497.00 | 4956.61 | 4378.06 | 4647.56 | 4641.20 |
| gene-evm.model.tig00000005_pilon_pilon_pilon.625 | 38.45 | 39.31 | 37.96 | 34.60 | 32.92 | 36.06 | 17.71 | 19.18 | 14.20 | 11.94 | 11.00 | 10.55 |
| gene-evm.model.tig00000005_pilon_pilon_pilon.624 | 1.14 | 1.84 | 1.48 | 0 | 0.22 | 0.22 | 0.19 | 0.45 | 0.95 | 0.13 | 0.082 | 0.38 |
| gene-evm.model.tig00000005_pilon_pilon_pilon.629 | 3.24 | 2.84 | 2.85 | 1.86 | 2.60 | 2.53 | 1.61 | 1.43 | 1.67 | 2.55 | 1.74 | 1.64 |

Note: #ID: gene ID; Values in the rest columns: FPKM value of the specific gene in each sample.

Result directory:

[BMK_7_Expression_Annotation/BMK_1_Expression_result](file:///C:\\Users\\27846\\Desktop\\%E8%AE%BA%E6%96%87\\%E9%9A%8B%E6%96%87%E9%9D%99\\BMK_7_Expression_Annotation\\BMK_1_Expression_result)

**9.2 Distribution of Gene Expression**

RNA-Seq is able to achieve highly-sensitive quantification of gene expression. Generally, a detectable trancriptome expression (FPKM) is ranging from 10^(-2) to 10^4[[19]](file:///C:\Users\27846\Desktop\%E8%AE%BA%E6%96%87\%E9%9A%8B%E6%96%87%E9%9D%99\index_en.html#ref19).

Fig14 FPKM density distribution of each sample

Note: Curves with different colours represent different samples; X-axis: log10(FPKM); Y-axis: Probabilty density.

The box plot presented the dispersion of gene expression within a sample and the comparison of overall expression among samples. The FPKM box plots of each sample was shown in the following figure.

Fig15 FPKM boxplot of each sample

Note: X-axis: Sample IDs; Y-axis: log10(FPKM); This plot shows the overall expression level of each sample via the dispersion of gene expression in each sample.

Result directory:

BMK_7_Expression_Annotation/BMK_2_Expression_distribution:

**9.3 Correlation assessment of biological replicates**

It has been widely proven that gene expression level fluctuates among individuals differently (known as biological variability)[[20]](file:///C:\Users\27846\Desktop\%E8%AE%BA%E6%96%87\%E9%9A%8B%E6%96%87%E9%9D%99\index_en.html#ref20)[[21]](file:///C:\Users\27846\Desktop\%E8%AE%BA%E6%96%87\%E9%9A%8B%E6%96%87%E9%9D%99\index_en.html#ref21), which can not be eliminated via RNA sequencing, qPCR or microarray. In order to identify genes with true differential expression between groups, biological variabliity should be taken into consideration[[22]](file:///C:\Users\27846\Desktop\%E8%AE%BA%E6%96%87\%E9%9A%8B%E6%96%87%E9%9D%99\index_en.html#ref22). To date, one of the most commonly used and effective method to distinguish random fluctuation and real difference is to design biological replicates. The reliability of differential expression analysis is largely depending on the quality and the number of replicates. Therefore, in projects with biological replicates, it is crucial to ensure the reproducibility of the replicates by correlation analysis. In addition, correlation analysis could also help screening for abnormal samples.

Pearson correlation coefficient R (Pearson's Correlation Coefficient) was applied in this project to evaluate reproducibility of biological replicates[[23]](file:///C:\Users\27846\Desktop\%E8%AE%BA%E6%96%87\%E9%9A%8B%E6%96%87%E9%9D%99\index_en.html#ref23). A closer R2 value to 1 indicates better reproducibility between the two samples. We committed that all biological replicates will be processed by the same technician in the same batch including RNA extraction and library construction. The libraries will be sequenced in the same run on the same lane. We will also perform in depth analysis on abnormal samples.Basing on the outputs, we will discuss with our clients and make final decision on whether the abnormal sample should be removed in downstream analysis.

Correlations between samples were shown below.

Fig16 Correlation heatmap between samples

Result directory:

[BMK_7_Expression_Annotation/BMK_3_Sample_correlation](file:///C:\\Users\\27846\\Desktop\\%E8%AE%BA%E6%96%87\\%E9%9A%8B%E6%96%87%E9%9D%99\\BMK_7_Expression_Annotation\\BMK_3_Sample_correlation)

**10 Differential Expression Analysis**

The expression of a gene can be influenced by both external stimuli and internal environment, which is highly temporal-specific and tissue-specific. The genes expressed significantly different under different conditions, such as treatment vs control, wild type vs mutants, different time points, different tissue, etc., are defined as Differentially Expressed Genes (DEG). Similarly, transcripts with significantly different expression level are named Differentially Expressed Transcript (DET). The collection of genes acquired in differential expression analysis is defined as DEG set. In result files, the gene sets were named as "A_VS_B" to specify the comparing pair. Normally, "A" represents control group, wild type or former time point. "B" normally represents corresponding treated group, mutant or later time point. The genes with a higher expression level in B than A are defined as up-regulated genes. The ones with lower expression level in B are defined as down-regulated genes. Therefore, up-reg and down-reg are relative definitions, which relies on the order of A and B.

For experiments with biological replicates, differential expression analysis is processed by DESeq2[[24]](file:///C:\Users\27846\Desktop\%E8%AE%BA%E6%96%87\%E9%9A%8B%E6%96%87%E9%9D%99\index_en.html#ref24). For projects without biological replicates, edgeR[[25]](file:///C:\\Users\\27846\\Desktop\\%E8%AE%BA%E6%96%87\\%E9%9A%8B%E6%96%87%E9%9D%99\\index_en.html#ref25) is applied.

Criteria for differentially expressed genes was set as Fold Change(FC)≥2 and FDR<0.01. Fold change(FC) refers to the ratio of gene expression in two samples. False Discovery Rate (FDR) refers to adjusted p-value, which is used to measure significancy of difference.

Differential expressed genes identified in all groups were shown below.

Table10 Statistics on DEGs

| **DEG Set** | **DEG Number** | **up-regulated** | **down-regulated** |
| --- | --- | --- | --- |
| A_vs_B | 1,918 | 756 | 1,162 |
| A_vs_C | 3,168 | 1,185 | 1,983 |
| A_vs_D | 5,006 | 2,296 | 2,710 |

Note: DEG Set: Comparing sample pair; DEG Number: Number of differentially expressed genes; up-regulated: Number of up-regulated genes; down-regulated: Number of down-regulated genes.

Statistics on DEGs directory:

[BMK_8_DEG_Analysis](file:///C:\\Users\\27846\\Desktop\\%E8%AE%BA%E6%96%87\\%E9%9A%8B%E6%96%87%E9%9D%99\\BMK_8_DEG_Analysis)

Differential expression analysis output are as follows.

Table11 Differential expression analysis output

| **#ID** | **A2_Count** | **A1_Count** | **A3_Count** | **B3_Count** | **B2_Count** | **B1_Count** | **A2_FPKM** | **A1_FPKM** | **A3_FPKM** | **B3_FPKM** | **B2_FPKM** | **B1_FPKM** | **FDR** | **log2FC** | **regulated** |
| --- | --- | --- | --- | --- | --- | --- | --- | --- | --- | --- | --- | --- | --- | --- | --- |
| gene-evm.model.tig0000001...... | 665 | 710 | 696 | 214 | 158 | 202 | 24.55 | 26.31 | 26.69 | 8.31 | 6.30 | 7.92 | 1.57098974211361e-27 | -1.7 | down |
| gene-evm.model.tig0000000...... | 94 | 92 | 60 | 40 | 25 | 34 | 1.53 | 1.51 | 1.02 | 0.67 | 0.44 | 0.58 | 0.0017 | -1.1 | down |
| gene-evm.model.tig0000000...... | 225 | 200 | 174 | 91 | 91 | 77 | 2.18 | 1.94 | 1.74 | 0.92 | 0.95 | 0.79 | 2.34148052980267e-06 | -1 | down |
| Pestalotiopsis_kenyana_newGene_418 | 119 | 124 | 105 | 46 | 51 | 39 | 1.07 | 1.12 | 0.98 | 0.43 | 0.49 | 0.37 | 1.88731245234732e-05 | -1.2 | down |

Note: ID: Gene ID; *_Count: Gene expression(reads count) in corresponding sample; *_FPKM: Gene expression(FPKM) in corresponding sample; FDR/Pvalue: False Discovery Rate/Pvalue; log2FC: Fold change normalized by log2; regulated: Up or down regulated.

Volcano plot is able to directly present difference in gene expression between two samples and statistical significancy of the difference. Volcano plots of two samples were shown below.

- A_vs_B.Volcano.png
- A_vs_C.Volcano.png
- A_vs_D.Volcano.png

Fig17 Volcano plot on differential expression

Note: In volcano plot, each dot represents a gene. X-axis: log2Fold change of expression; Y-axis: -log10(FDR) or -log10(P-value). Dots farther to y=0 represent genes with large difference in expression between two samples. Dots farther to x=0 represents genes of which the difference is more reliable. Green dots are down-regulated genes, while red dots are up-regulated ones and black dots are genes without significant difference.

A plots shows the overall distribution of gene expression and fold change of expression level between two samples. MA plot of differentially expressed genes were shown in figures below.

- A_vs_B.MA.png
- A_vs_C.MA.png
- A_vs_D.MA.png

Fig18 MA plot of differentially expressed genes

Note: In MA plot, each dot represents a single gene.
X-axis: A value, i.e. log2 (FPKM);
Y-axis: M value, i.e. log2(FC);
The dots coloured in red and green stand for significant up-regulated and down-regulated genes respectively. Black dots stand for the genes without significant difference in expression between two samples.

Differential expression analysis output directory:

[BMK_8_DEG_Analysis/BMK_1_A_vs_B/BMK_1_DEG_stat](file:///C:\\Users\\27846\\Desktop\\%E8%AE%BA%E6%96%87\\%E9%9A%8B%E6%96%87%E9%9D%99\\BMK_8_DEG_Analysis\\BMK_1_A_vs_B\\BMK_1_DEG_stat)

[BMK_8_DEG_Analysis/BMK_1_A_vs_C/BMK_1_DEG_stat](file:///C:\\Users\\27846\\Desktop\\%E8%AE%BA%E6%96%87\\%E9%9A%8B%E6%96%87%E9%9D%99\\BMK_8_DEG_Analysis\\BMK_1_A_vs_C\\BMK_1_DEG_stat)

[BMK_8_DEG_Analysis/BMK_1_A_vs_D/BMK_1_DEG_stat](file:///C:\\Users\\27846\\Desktop\\%E8%AE%BA%E6%96%87\\%E9%9A%8B%E6%96%87%E9%9D%99\\BMK_8_DEG_Analysis\\BMK_1_A_vs_D\\BMK_1_DEG_stat)

Hierarchical clustering analysis was processed on differentially expressed gene, i.e. genes with same or similar expression mode were clustered together.

- A_vs_B.png
- A_vs_C.png
- A_vs_D.png

Fig19 Hierarchical clustering of differentially expressed genes

Note: Each column represents one sample and rows represent genes.
The expression level of genes (FPKM) was normalized by log10, i.e. log10（FPKM+0.000001）and presented as different colours based on scale bar.

Hierarchical clustering of differentially expressed genes directory:

[BMK_8_DEG_Analysis/BMK_1_A_vs_B/BMK_2_DEG_Cluster](file:///C:\\Users\\27846\\Desktop\\%E8%AE%BA%E6%96%87\\%E9%9A%8B%E6%96%87%E9%9D%99\\BMK_8_DEG_Analysis\\BMK_1_A_vs_B\\BMK_2_DEG_Cluster)

[BMK_8_DEG_Analysis/BMK_1_A_vs_C/BMK_2_DEG_Cluster](file:///C:\\Users\\27846\\Desktop\\%E8%AE%BA%E6%96%87\\%E9%9A%8B%E6%96%87%E9%9D%99\\BMK_8_DEG_Analysis\\BMK_1_A_vs_C\\BMK_2_DEG_Cluster)

[BMK_8_DEG_Analysis/BMK_1_A_vs_D/BMK_2_DEG_Cluster](file:///C:\\Users\\27846\\Desktop\\%E8%AE%BA%E6%96%87\\%E9%9A%8B%E6%96%87%E9%9D%99\\BMK_8_DEG_Analysis\\BMK_1_A_vs_D\\BMK_2_DEG_Cluster)

Venn diagram of differentially expressed genes between samples was shown in the figure below. Venn diagram shows the number of unique DEGs between each comparing pair and overlapping of DEGs between different comparing pairs.

Fig20 Venn diagram of differentially expressed genes

Venn diagram of differentially expressed genes directory:

[BMK_8_DEG_Analysis/BMK_2_All_DEG](file:///C:\\Users\\27846\\Desktop\\%E8%AE%BA%E6%96%87\\%E9%9A%8B%E6%96%87%E9%9D%99\\BMK_8_DEG_Analysis\\BMK_2_All_DEG)

**11 Enrichment Analysis of DEGs**

The DEGs identified in differential expression analysis were annotated. The summary of annotations was shown in the table below.

Table12 Summary of DEGs annotation

| **DEG Set** | **Total** | **COG** | **GO** | **KEGG** | **KOG** | **NR** | **Pfam** | **Swiss-Prot** | **eggNOG** |
| --- | --- | --- | --- | --- | --- | --- | --- | --- | --- |
| A_vs_B | 1,570 | 746 | 1,097 | 941 | 610 | 1,567 | 1,175 | 841 | 1,172 |
| A_vs_C | 2,638 | 1,238 | 1,787 | 1,496 | 912 | 2,634 | 1,952 | 1,307 | 1,937 |
| A_vs_D | 4,289 | 2,065 | 3,013 | 2,550 | 1,716 | 4,282 | 3,277 | 2,242 | 3,242 |

Note: DEG Set: Group set of DEG analysis; Total: Number of annotated DEGs; The rest columns are the numbers of annotated DEGs in corresponding database.

**11.1 COG Classification on DEGs**

COG（Cluster of Orthologous Groups of proteins is a database collecting phylogenetic classification of proteins, which can be provide orthologous classification information of gene products.

Summary of COG classifications on DEGs were shown in the figures below.

- A_vs_B.COG.classify.png
- A_vs_C.COG.classify.png
- A_vs_D.COG.classify.png

Fig21 Summary of COG classifications on DEGs

Note: X-axis: COG classification terms; Y-axis: Number of genes in the term.
In the different functional classes, the number of genes reflects the preference of gene functions in different experimental groups, such as metabolic function or physiological bias, etc. These can be explained based on specific research subjects.

Summary of COG classifications on DEGs directory:

[BMK_8_DEG_Analysis/BMK_1_A_vs_B/BMK_3_Anno_enrichment/BMK_1_All_enrichment/BMK_1_DEG_Anno](file:///C:\\Users\\27846\\Desktop\\%E8%AE%BA%E6%96%87\\%E9%9A%8B%E6%96%87%E9%9D%99\\BMK_8_DEG_Analysis\\BMK_1_A_vs_B\\BMK_3_Anno_enrichment\\BMK_1_All_enrichment\\BMK_1_DEG_Anno)

[BMK_8_DEG_Analysis/BMK_1_A_vs_C/BMK_3_Anno_enrichment/BMK_1_All_enrichment/BMK_1_DEG_Anno](file:///C:\\Users\\27846\\Desktop\\%E8%AE%BA%E6%96%87\\%E9%9A%8B%E6%96%87%E9%9D%99\\BMK_8_DEG_Analysis\\BMK_1_A_vs_C\\BMK_3_Anno_enrichment\\BMK_1_All_enrichment\\BMK_1_DEG_Anno)

[BMK_8_DEG_Analysis/BMK_1_A_vs_D/BMK_3_Anno_enrichment/BMK_1_All_enrichment/BMK_1_DEG_Anno](file:///C:\\Users\\27846\\Desktop\\%E8%AE%BA%E6%96%87\\%E9%9A%8B%E6%96%87%E9%9D%99\\BMK_8_DEG_Analysis\\BMK_1_A_vs_D\\BMK_3_Anno_enrichment\\BMK_1_All_enrichment\\BMK_1_DEG_Anno)

**11.2 GO Analysis on DEGs**

GO (Gene Ontology) database is a structured biological annotation system established in 2000 containing a standard vocabulary of gene and gene products functions. GO annotation system is a directed acyclic graph containing three main branches: Biological Process, Molecular Function and Cellular Component.

GO classification of DEGs between samples was shown in the following figures.

- A_vs_B.GO_classification.png
- A_vs_C.GO_classification.png
- A_vs_D.GO_classification.png

Fig22 GO classification of DEGs

Note: X-axis: Go terms and classifications; Y-axis: Number of DEGs(genes) annotated to the term(right) and percentage of that in all DEGs(genes) (Left).This figure shows the GO enrichment in DEGs and in all genes, which indicates the importance of a specific GO term in DEGs and all genes respectively. The terms with two bars significantly different from each other can be picked up as potential targets for further analysis on functions, since these GO terms are enriched differently between DEGs-based and all-gene-based enrichment.

GO Analysis on DEGs directory:

[BMK_8_DEG_Analysis/BMK_1_A_vs_B/BMK_3_Anno_enrichment/BMK_1_All_enrichment/BMK_2_GO_enrichment](file:///C:\\Users\\27846\\Desktop\\%E8%AE%BA%E6%96%87\\%E9%9A%8B%E6%96%87%E9%9D%99\\BMK_8_DEG_Analysis\\BMK_1_A_vs_B\\BMK_3_Anno_enrichment\\BMK_1_All_enrichment\\BMK_2_GO_enrichment)

[BMK_8_DEG_Analysis/BMK_1_A_vs_C/BMK_3_Anno_enrichment/BMK_1_All_enrichment/BMK_2_GO_enrichment](file:///C:\\Users\\27846\\Desktop\\%E8%AE%BA%E6%96%87\\%E9%9A%8B%E6%96%87%E9%9D%99\\BMK_8_DEG_Analysis\\BMK_1_A_vs_C\\BMK_3_Anno_enrichment\\BMK_1_All_enrichment\\BMK_2_GO_enrichment)

[BMK_8_DEG_Analysis/BMK_1_A_vs_D/BMK_3_Anno_enrichment/BMK_1_All_enrichment/BMK_2_GO_enrichment](file:///C:\\Users\\27846\\Desktop\\%E8%AE%BA%E6%96%87\\%E9%9A%8B%E6%96%87%E9%9D%99\\BMK_8_DEG_Analysis\\BMK_1_A_vs_D\\BMK_3_Anno_enrichment\\BMK_1_All_enrichment\\BMK_2_GO_enrichment)

**11.3 GO Enrichment Analysis on DEGs**

DEGs were then subjected to functional enrichment analysis and the enriched GO terms and corresponding inclusion relationships were shown in the directed acyclic graph. In the figure, the direction of arrows represents inclusion relations between terms, i.e. the nodes are more specific than their upper nodes. Directed acyclic graphs of DEGs generated by topGO[[26]](file:///C:\\Users\\27846\\Desktop\\%E8%AE%BA%E6%96%87\\%E9%9A%8B%E6%96%87%E9%9D%99\\index_en.html#ref26) were shown below.

- A_vs_B.topGO_BP.png
- A_vs_B.topGO_CC.png
- A_vs_B.topGO_MF.png
- A_vs_C.topGO_BP.png
- A_vs_C.topGO_CC.png
- A_vs_C.topGO_MF.png
- A_vs_D.topGO_BP.png
- A_vs_D.topGO_CC.png
- A_vs_D.topGO_MF.png

Fig23 TopGO directed acyclic graph of DEGs

Note: The most significantly enriched 10 terms were shown in cubes. Each box or node contains a description of GO term and significancy value of enrichment. The colour represents the significancy, where a darker colour indicates a more significant enrichment.

GO Enrichment Analysis on DEGs directory:

[BMK_8_DEG_Analysis/BMK_1_A_vs_B/BMK_3_Anno_enrichment/BMK_1_All_enrichment/BMK_2_GO_enrichment](file:///C:\\Users\\27846\\Desktop\\%E8%AE%BA%E6%96%87\\%E9%9A%8B%E6%96%87%E9%9D%99\\BMK_8_DEG_Analysis\\BMK_1_A_vs_B\\BMK_3_Anno_enrichment\\BMK_1_All_enrichment\\BMK_2_GO_enrichment)

[BMK_8_DEG_Analysis/BMK_1_A_vs_C/BMK_3_Anno_enrichment/BMK_1_All_enrichment/BMK_2_GO_enrichment](file:///C:\\Users\\27846\\Desktop\\%E8%AE%BA%E6%96%87\\%E9%9A%8B%E6%96%87%E9%9D%99\\BMK_8_DEG_Analysis\\BMK_1_A_vs_C\\BMK_3_Anno_enrichment\\BMK_1_All_enrichment\\BMK_2_GO_enrichment)

[BMK_8_DEG_Analysis/BMK_1_A_vs_D/BMK_3_Anno_enrichment/BMK_1_All_enrichment/BMK_2_GO_enrichment](file:///C:\\Users\\27846\\Desktop\\%E8%AE%BA%E6%96%87\\%E9%9A%8B%E6%96%87%E9%9D%99\\BMK_8_DEG_Analysis\\BMK_1_A_vs_D\\BMK_3_Anno_enrichment\\BMK_1_All_enrichment\\BMK_2_GO_enrichment)

**11.4 KEGG annotation of differentially expressed genes**

In biological organisms, series of gene products are working synergistically to perform biological functions, which is so called pathway. Annotating genes within pathway networks could largely benefit further analysis on biological functions. KEGG (Kyoto Encyclopedia of Genes and Genomes) is one of the major databases on pathways, including metabolic pathways of carbohydrates, nucleotides, amino acids and biological degradation of organics. Besides metabolic pathways, KEGG contains comprehensive description on enzymes involved in the pathways, including amino acid sequences, links to PDB database, etc.

KEGG pathway annotation on DEGs example is shown in the following figure.

Fig24 DEMO of KEGG annotation on DEGs

Note: Relative to control group, the nodes coloured in red represent the enzymes related to up-regulated genes and the green ones represent that of down-regulated genes.
Blue ones represent enzymes related to both up and down-regulated genes. The number in the box stands for EC number. The pathway consists of many complex biochemical reactions involving multiple enzymes. The DEGs annotated to the pathway were coloured on the figure. Researchers can pick pathways of their own interest basing on the highlighted pathways and research subjects for further analysis and interpretation.

The KEGG annotations of DEGs were classified according to the type of pathways. Detailed classification was shown in the following figure.

- A_vs_B.KEGG_classification.png
- A_vs_C.KEGG_classification.png
- A_vs_D.KEGG_classification.png

Fig25 KEGG Classification on DEGs

Note: Y-axis: KEGG pathway terms; X-axis: Number and the percentage of genes annotated to the KEGG pathway.

KEGG annotation of DEGs directory:

[BMK_8_DEG_Analysis/BMK_1_A_vs_B/BMK_3_Anno_enrichment/BMK_1_All_enrichment/BMK_3_KEGG_enrichment](file:///C:\\Users\\27846\\Desktop\\%E8%AE%BA%E6%96%87\\%E9%9A%8B%E6%96%87%E9%9D%99\\BMK_8_DEG_Analysis\\BMK_1_A_vs_B\\BMK_3_Anno_enrichment\\BMK_1_All_enrichment\\BMK_3_KEGG_enrichment)

[BMK_8_DEG_Analysis/BMK_1_A_vs_C/BMK_3_Anno_enrichment/BMK_1_All_enrichment/BMK_3_KEGG_enrichment](file:///C:\\Users\\27846\\Desktop\\%E8%AE%BA%E6%96%87\\%E9%9A%8B%E6%96%87%E9%9D%99\\BMK_8_DEG_Analysis\\BMK_1_A_vs_C\\BMK_3_Anno_enrichment\\BMK_1_All_enrichment\\BMK_3_KEGG_enrichment)

[BMK_8_DEG_Analysis/BMK_1_A_vs_D/BMK_3_Anno_enrichment/BMK_1_All_enrichment/BMK_3_KEGG_enrichment](file:///C:\\Users\\27846\\Desktop\\%E8%AE%BA%E6%96%87\\%E9%9A%8B%E6%96%87%E9%9D%99\\BMK_8_DEG_Analysis\\BMK_1_A_vs_D\\BMK_3_Anno_enrichment\\BMK_1_All_enrichment\\BMK_3_KEGG_enrichment)

**11.5 KEGG Pathway enrichment analysis on DEGs**

In this session, we examined if the pathways are over-presented with DEGs. Enrichment factors and fisher test were applied in the determination of enrichment degree and significancy of the pathway. Enrichment of DEGs in KEGG pathways are shown in the figures below. Top 20 enriched pathways (with smallest Q-value) were shown.

- A_vs_B_KEGG_enrichment.png
- A_vs_C_KEGG_enrichment.png
- A_vs_D_KEGG_enrichment.png

Fig26 KEGG pathway enrichment on DEGs-Bubble chart

Note: Each dot represents a KEGG pathway. Y-axis: Pathway; X-axis: Enrichment factor. Enrichment factor is calculated as "Enrichment factor=(Ratio of DEGs annotated to the term over all DEGs)/(Ratio of genes annotated to the term over all genes)"
A larger enrichment factor indicates a more significant enrichment of the pathway.
The colour of the dots stands for q-value (adjusted p-value). The smaller the q-value is, the more significant or reliable the enrichment is.
The size of the dots represents the number of DEGs enriched in this pathway. The larger the dot is, the more genes it contains.

In this figure, the dots closer to upper right area are more reliable in differential analysis. Top 20 enriched pathways (with smallest Q-value) were shown.

KEGG enrichment analysis on DEGs directory:

[BMK_8_DEG_Analysis/BMK_1_A_vs_B/BMK_3_Anno_enrichment/BMK_1_All_enrichment/BMK_3_KEGG_enrichment](file:///C:\\Users\\27846\\Desktop\\%E8%AE%BA%E6%96%87\\%E9%9A%8B%E6%96%87%E9%9D%99\\BMK_8_DEG_Analysis\\BMK_1_A_vs_B\\BMK_3_Anno_enrichment\\BMK_1_All_enrichment\\BMK_3_KEGG_enrichment)

[BMK_8_DEG_Analysis/BMK_1_A_vs_C/BMK_3_Anno_enrichment/BMK_1_All_enrichment/BMK_3_KEGG_enrichment](file:///C:\\Users\\27846\\Desktop\\%E8%AE%BA%E6%96%87\\%E9%9A%8B%E6%96%87%E9%9D%99\\BMK_8_DEG_Analysis\\BMK_1_A_vs_C\\BMK_3_Anno_enrichment\\BMK_1_All_enrichment\\BMK_3_KEGG_enrichment)

[BMK_8_DEG_Analysis/BMK_1_A_vs_D/BMK_3_Anno_enrichment/BMK_1_All_enrichment/BMK_3_KEGG_enrichment](file:///C:\\Users\\27846\\Desktop\\%E8%AE%BA%E6%96%87\\%E9%9A%8B%E6%96%87%E9%9D%99\\BMK_8_DEG_Analysis\\BMK_1_A_vs_D\\BMK_3_Anno_enrichment\\BMK_1_All_enrichment\\BMK_3_KEGG_enrichment)

**11.6 GSEA analysis**

Gene Set Enrichment Analysis(GSEA)[[32]](file:///C:\Users\27846\Desktop\%E8%AE%BA%E6%96%87\%E9%9A%8B%E6%96%87%E9%9D%99\index_en.html#ref32) was processed on all genes based on expression level. Normally, differential expression analysis only focus on up- or down-regulated genes with statistical significancy. However, this may mask the genes, which are altered slightly without significancy but play vital role in biological functions. Without setting threshold on fold change and significancy, GSEA is able to detect weak alterations in gene expression. In this analysis, genes sets of KEGG pathway and GO terms on BP, CC, MF were employed as gene sets of interest. Genes of each group were used as background gene set. Enriched gene sets were identified as p-value<0.001 and FDR<0.05.

- A_vs_B_Biological_Process_GO_0006396.gseaplot.png
- A_vs_B_Biological_Process_GO_0006412.gseaplot.png
- A_vs_B_Biological_Process_GO_0016070.gseaplot.png
- A_vs_B_Biological_Process_GO_0022613.gseaplot.png
- A_vs_B_Biological_Process_GO_0042254.gseaplot.png
- A_vs_B_Cellular_Component_GO_0005730.gseaplot.png
- A_vs_B_Cellular_Component_GO_0031974.gseaplot.png
- A_vs_B_Cellular_Component_GO_0031981.gseaplot.png
- A_vs_B_Cellular_Component_GO_0043233.gseaplot.png
- A_vs_B_Cellular_Component_GO_0070013.gseaplot.png
- A_vs_B_KEGG_pathway_ko00562.gseaplot.png
- A_vs_B_KEGG_pathway_ko03010.gseaplot.png
- A_vs_B_KEGG_pathway_ko03020.gseaplot.png
- A_vs_B_KEGG_pathway_ko03050.gseaplot.png
- A_vs_B_KEGG_pathway_ko04138.gseaplot.png
- A_vs_B_Molecular_Function_GO_0003676.gseaplot.png
- A_vs_B_Molecular_Function_GO_0003723.gseaplot.png
- A_vs_B_Molecular_Function_GO_0003735.gseaplot.png
- A_vs_B_Molecular_Function_GO_0004386.gseaplot.png
- A_vs_B_Molecular_Function_GO_0030515.gseaplot.png
- A_vs_C_Biological_Process_GO_0000463.gseaplot.png
- A_vs_C_Biological_Process_GO_0001732.gseaplot.png
- A_vs_C_Biological_Process_GO_0006351.gseaplot.png
- A_vs_C_Biological_Process_GO_0007010.gseaplot.png
- A_vs_C_Biological_Process_GO_0030071.gseaplot.png
- A_vs_C_Cellular_Component_GO_0005856.gseaplot.png
- A_vs_C_Cellular_Component_GO_0016282.gseaplot.png
- A_vs_C_Cellular_Component_GO_0030688.gseaplot.png
- A_vs_C_Cellular_Component_GO_0033290.gseaplot.png
- A_vs_C_Cellular_Component_GO_0042025.gseaplot.png
- A_vs_C_KEGG_pathway_ko03010.gseaplot.png
- A_vs_C_KEGG_pathway_ko03015.gseaplot.png
- A_vs_C_KEGG_pathway_ko03050.gseaplot.png
- A_vs_C_KEGG_pathway_ko04138.gseaplot.png
- A_vs_C_KEGG_pathway_ko04145.gseaplot.png
- A_vs_C_Molecular_Function_GO_0000981.gseaplot.png
- A_vs_C_Molecular_Function_GO_0004497.gseaplot.png
- A_vs_C_Molecular_Function_GO_0005506.gseaplot.png
- A_vs_C_Molecular_Function_GO_0016705.gseaplot.png
- A_vs_C_Molecular_Function_GO_0020037.gseaplot.png
- A_vs_D_Biological_Process_GO_0006364.gseaplot.png
- A_vs_D_Biological_Process_GO_0006511.gseaplot.png
- A_vs_D_Biological_Process_GO_0050790.gseaplot.png
- A_vs_D_Biological_Process_GO_0051172.gseaplot.png
- A_vs_D_Biological_Process_GO_0051336.gseaplot.png
- A_vs_D_Cellular_Component_GO_0005730.gseaplot.png
- A_vs_D_Cellular_Component_GO_0005743.gseaplot.png
- A_vs_D_Cellular_Component_GO_0005840.gseaplot.png
- A_vs_D_Cellular_Component_GO_0005844.gseaplot.png
- A_vs_D_Cellular_Component_GO_0030688.gseaplot.png
- A_vs_D_KEGG_pathway_ko00053.gseaplot.png
- A_vs_D_KEGG_pathway_ko00220.gseaplot.png
- A_vs_D_KEGG_pathway_ko00562.gseaplot.png
- A_vs_D_KEGG_pathway_ko03050.gseaplot.png
- A_vs_D_KEGG_pathway_ko04141.gseaplot.png
- A_vs_D_Molecular_Function_GO_0003735.gseaplot.png
- A_vs_D_Molecular_Function_GO_0004252.gseaplot.png
- A_vs_D_Molecular_Function_GO_0004672.gseaplot.png
- A_vs_D_Molecular_Function_GO_0022857.gseaplot.png
- A_vs_D_Molecular_Function_GO_0030234.gseaplot.png

Fig27 GSEA enrichment plot

Note: In the upper figure, X-axis: Position of gene set after ordering; Y-axis: Enrichment score; The lines on the top represent genes in the gene set. Green curve shows the enrichment score of each gene set across positions. In the lower figure, X-axis: Position of gene set after ordering. Y-axis: Score. Each line represents a gene in gene set. The length of lines indicates corresponding score.

GSEA analysis directory:

[BMK_8_DEG_Analysis/BMK_1_A_vs_B/BMK_4_GSEA](file:///C:\\Users\\27846\\Desktop\\%E8%AE%BA%E6%96%87\\%E9%9A%8B%E6%96%87%E9%9D%99\\BMK_8_DEG_Analysis\\BMK_1_A_vs_B\\BMK_4_GSEA)

[BMK_8_DEG_Analysis/BMK_1_A_vs_C/BMK_4_GSEA](file:///C:\\Users\\27846\\Desktop\\%E8%AE%BA%E6%96%87\\%E9%9A%8B%E6%96%87%E9%9D%99\\BMK_8_DEG_Analysis\\BMK_1_A_vs_C\\BMK_4_GSEA)

[BMK_8_DEG_Analysis/BMK_1_A_vs_D/BMK_4_GSEA](file:///C:\\Users\\27846\\Desktop\\%E8%AE%BA%E6%96%87\\%E9%9A%8B%E6%96%87%E9%9D%99\\BMK_8_DEG_Analysis\\BMK_1_A_vs_D\\BMK_4_GSEA)

**11.7 Protein-protein Interaction Network of DEGs**

STRING[[27]](file:///C:\Users\27846\Desktop\%E8%AE%BA%E6%96%87\%E9%9A%8B%E6%96%87%E9%9D%99\index_en.html#ref27) is a database containing information of predicted and proved protein-protein interactions (PPI) of a collection of species. The interactions refer to both direct physical interactions and indirect functional interactions. The PPI network was built based on the DEGs generated in the differential expression analysis and existing information on interactions in database. For the species included in the database, the interactions of targeted genes can be extracted directly from the database for network construction. For species couldn't be found in STRING, homologous proteins were used for network construction. The PPT networks can be visualized by Cytoscape[[28]](file:///C:\Users\27846\Desktop\%E8%AE%BA%E6%96%87\%E9%9A%8B%E6%96%87%E9%9D%99\index_en.html#ref28).

PPI networks of DEGs visualized in Cytoscape were shown as below.

- P26_pp_network.png

Fig28 Protein-protein interaction network of DEGs

Note: Each node in the figure represents a protein. The edge between nodes represents interactions.
The size of the nodes represents their degree, i.e. the number of interactions linked to them. The larger the nodes are, the more interactions they are involved in.
The colour of the node is related to the clustering coefficient. With spectrum from green to red, the clustering coefficient increases. A higher clustering coefficient (red nodes) indicates a better connectivity of the node to surrounding nodes.
The thickness of the edge between two nodes represents the strength of interactions. The thicker the edge is, the stronger the interaction is.
Nodes without connections means there are no PPT found in the analysis.

Protein-protein Interaction Network of DEGs directory:

[BMK_8_DEG_Analysis/BMK_1_A_vs_B/BMK_5_PPI_String](file:///C:\\Users\\27846\\Desktop\\%E8%AE%BA%E6%96%87\\%E9%9A%8B%E6%96%87%E9%9D%99\\BMK_8_DEG_Analysis\\BMK_1_A_vs_B\\BMK_5_PPI_String)

[BMK_8_DEG_Analysis/BMK_1_A_vs_C/BMK_5_PPI_String](file:///C:\\Users\\27846\\Desktop\\%E8%AE%BA%E6%96%87\\%E9%9A%8B%E6%96%87%E9%9D%99\\BMK_8_DEG_Analysis\\BMK_1_A_vs_C\\BMK_5_PPI_String)

[BMK_8_DEG_Analysis/BMK_1_A_vs_D/BMK_5_PPI_String](file:///C:\\Users\\27846\\Desktop\\%E8%AE%BA%E6%96%87\\%E9%9A%8B%E6%96%87%E9%9D%99\\BMK_8_DEG_Analysis\\BMK_1_A_vs_D\\BMK_5_PPI_String)

**12 Differential Alternative Splicing Analysis**

Differential alternative splicing analysis was processed by rMATS[[31]](file:///C:\\Users\\27846\\Desktop\\%E8%AE%BA%E6%96%87\\%E9%9A%8B%E6%96%87%E9%9D%99\\index_en.html#ref31). The number of reads that uniquely mapped to the transcripte (the exon inclusion isoform or the exon skipping isoform) is defined as inclusion level of alternative splicing.The rMATS statistical calculates the p-value between IncLevel (Inclusion level) of two groups of samples by likelihood-ratio test. The p-values were then corrected by Benjamini Hochberg to get FDR value. In current analysis, the default threshold for rMATS screening is |Δψ| > c (c=0.0001); i.e. P-value between mean ψ values of two samples larger than the threshold c. rMATS can identify following 5 alternative splicing events: exon skipping (SE), alternative 5' splicing junction(A5SS), alternative 3' splicing junction (A3SS), mutually exclusive exon (MXE) and intron retention (RI).

rMATS software analysis results:

1. [A_vs_B.A3SS.MATS.JC.xls](file:///C:\Users\27846\Desktop\%E8%AE%BA%E6%96%87\%E9%9A%8B%E6%96%87%E9%9D%99\BMK_8_DEG_Analysis\BMK_1_A_vs_B\BMK_6_diff_AS_analysis\A3SS.MATS.JC.xls)
2. [A_vs_B.A5SS.MATS.JC.xls](file:///C:\Users\27846\Desktop\%E8%AE%BA%E6%96%87\%E9%9A%8B%E6%96%87%E9%9D%99\BMK_8_DEG_Analysis\BMK_1_A_vs_B\BMK_6_diff_AS_analysis\A5SS.MATS.JC.xls)
3. [A_vs_B.MXE.MATS.JC.xls](file:///C:\Users\27846\Desktop\%E8%AE%BA%E6%96%87\%E9%9A%8B%E6%96%87%E9%9D%99\BMK_8_DEG_Analysis\BMK_1_A_vs_B\BMK_6_diff_AS_analysis\MXE.MATS.JC.xls)
4. [A_vs_B.RI.MATS.JC.xls](file:///C:\Users\27846\Desktop\%E8%AE%BA%E6%96%87\%E9%9A%8B%E6%96%87%E9%9D%99\BMK_8_DEG_Analysis\BMK_1_A_vs_B\BMK_6_diff_AS_analysis\RI.MATS.JC.xls)
5. [A_vs_B.SE.MATS.JC.xls](file:///C:\Users\27846\Desktop\%E8%AE%BA%E6%96%87\%E9%9A%8B%E6%96%87%E9%9D%99\BMK_8_DEG_Analysis\BMK_1_A_vs_B\BMK_6_diff_AS_analysis\SE.MATS.JC.xls)
6. [A_vs_C.A3SS.MATS.JC.xls](file:///C:\Users\27846\Desktop\%E8%AE%BA%E6%96%87\%E9%9A%8B%E6%96%87%E9%9D%99\BMK_8_DEG_Analysis\BMK_1_A_vs_C\BMK_6_diff_AS_analysis\A3SS.MATS.JC.xls)
7. [A_vs_C.A5SS.MATS.JC.xls](file:///C:\Users\27846\Desktop\%E8%AE%BA%E6%96%87\%E9%9A%8B%E6%96%87%E9%9D%99\BMK_8_DEG_Analysis\BMK_1_A_vs_C\BMK_6_diff_AS_analysis\A5SS.MATS.JC.xls)
8. [A_vs_C.MXE.MATS.JC.xls](file:///C:\Users\27846\Desktop\%E8%AE%BA%E6%96%87\%E9%9A%8B%E6%96%87%E9%9D%99\BMK_8_DEG_Analysis\BMK_1_A_vs_C\BMK_6_diff_AS_analysis\MXE.MATS.JC.xls)
9. [A_vs_C.RI.MATS.JC.xls](file:///C:\Users\27846\Desktop\%E8%AE%BA%E6%96%87\%E9%9A%8B%E6%96%87%E9%9D%99\BMK_8_DEG_Analysis\BMK_1_A_vs_C\BMK_6_diff_AS_analysis\RI.MATS.JC.xls)
10. [A_vs_C.SE.MATS.JC.xls](file:///C:\Users\27846\Desktop\%E8%AE%BA%E6%96%87\%E9%9A%8B%E6%96%87%E9%9D%99\BMK_8_DEG_Analysis\BMK_1_A_vs_C\BMK_6_diff_AS_analysis\SE.MATS.JC.xls)
11. [A_vs_D.A3SS.MATS.JC.xls](file:///C:\Users\27846\Desktop\%E8%AE%BA%E6%96%87\%E9%9A%8B%E6%96%87%E9%9D%99\BMK_8_DEG_Analysis\BMK_1_A_vs_D\BMK_6_diff_AS_analysis\A3SS.MATS.JC.xls)
12. [A_vs_D.A5SS.MATS.JC.xls](file:///C:\Users\27846\Desktop\%E8%AE%BA%E6%96%87\%E9%9A%8B%E6%96%87%E9%9D%99\BMK_8_DEG_Analysis\BMK_1_A_vs_D\BMK_6_diff_AS_analysis\A5SS.MATS.JC.xls)
13. [A_vs_D.MXE.MATS.JC.xls](file:///C:\Users\27846\Desktop\%E8%AE%BA%E6%96%87\%E9%9A%8B%E6%96%87%E9%9D%99\BMK_8_DEG_Analysis\BMK_1_A_vs_D\BMK_6_diff_AS_analysis\MXE.MATS.JC.xls)
14. [A_vs_D.RI.MATS.JC.xls](file:///C:\Users\27846\Desktop\%E8%AE%BA%E6%96%87\%E9%9A%8B%E6%96%87%E9%9D%99\BMK_8_DEG_Analysis\BMK_1_A_vs_D\BMK_6_diff_AS_analysis\RI.MATS.JC.xls)
15. [A_vs_D.SE.MATS.JC.xls](file:///C:\Users\27846\Desktop\%E8%AE%BA%E6%96%87\%E9%9A%8B%E6%96%87%E9%9D%99\BMK_8_DEG_Analysis\BMK_1_A_vs_D\BMK_6_diff_AS_analysis\SE.MATS.JC.xls)

Table13 Differential alternative splicing analysis (SE splicing as an example)

| **GeneID** | **chr** | **strand** | **exonStart_0base** | **exonEnd** | **upstreamES** | **upstreamEE** | **downstreamES** | **downstreamEE** | **ID** | **IJC_SAMPLE_1** | **SJC_SAMPLE_1** | **IJC_SAMPLE_2** | **SJC_SAMPLE_2** | **IncFormLen** | **SkipFormLen** | **PValue** | **FDR** | **IncLevel1** | **IncLevel2** | **IncLevelDifference** |
| --- | --- | --- | --- | --- | --- | --- | --- | --- | --- | --- | --- | --- | --- | --- | --- | --- | --- | --- | --- | --- |
| gene-evm.model.tig00000031_pilon_pilon_pilon.1457 | tig00000031_pilon_pilon_pilon | - | 4,415,970 | 4,416,201 | 4,415,795 | 4,415,845 | 4,416,429 | 4,416,483 | 0 | 2272,2290,2302 | 0,0,0 | 2323,2242,2565 | 1,0,0 | 298 | 149 | 1 | 1 | 1.0,1.0,1.0 | 0.999,1.0,1.0 | 0 |
| gene-evm.model.tig00000005_pilon_pilon_pilon.1364 | tig00000005_pilon_pilon_pilon | + | 4,252,750 | 4,252,843 | 4,252,432 | 4,252,660 | 4,252,907 | 4,252,954 | 1 | 150,203,179 | 1,0,0 | 193,151,150 | 0,0,0 | 241 | 149 | 1 | 1 | 0.989,1.0,1.0 | 1.0,1.0,1.0 | -0.004 |
| gene-evm.model.tig00000031_pilon_pilon_pilon.1392 | tig00000031_pilon_pilon_pilon | - | 4,207,340 | 4,207,415 | 4,206,729 | 4,206,971 | 4,207,626 | 4,207,654 | 2 | 2615,2907,2991 | 0,0,0 | 3889,3935,3588 | 1,6,0 | 223 | 149 | 1 | 1 | 1.0,1.0,1.0 | 1.0,0.998,1.0 | 0.001 |
| gene-evm.model.tig00000007_pilon_pilon_pilon.427 | tig00000007_pilon_pilon_pilon | - | 1,338,198 | 1,338,630 | 1,337,968 | 1,338,133 | 1,338,725 | 1,338,815 | 3 | 7,8,7 | 0,0,0 | 7,8,6 | 1,0,0 | 298 | 149 | 1 | 1 | 1.0,1.0,1.0 | 0.778,1.0,1.0 | 0.074 |
| gene-evm.model.tig00000010_pilon_pilon_pilon.1675 | tig00000010_pilon_pilon_pilon | - | 5,438,608 | 5,438,743 | 5,438,045 | 5,438,515 | 5,438,817 | 5,438,929 | 4 | 258,159,226 | 2,1,0 | 143,171,172 | 0,0,0 | 283 | 149 | 1 | 1 | 0.985,0.988,1.0 | 1.0,1.0,1.0 | -0.009 |

Note: GeneID: Gene ID; geneSymbol: Gene symbol; chr: chromosome No. Strand: +/-strand; exonStart_0base: Starting position of exon (from 0); exonEnd: Ending position of exon; upstreamES: Starting position of upper-stream exon; upstreamEE: Ending position of upper-stream exon; downstreamES: Starting position of down-stream exon; downstreamEE: Ending position of downstream exon; (Other types of alternative splicing may have some different columns); IJC_SAMPLE_1：counts of inclusion junction counts in SAMPLE_1, replicates are divided by ","; SJC_SAMPLE_1： counts of skipping junction in SAMPLE_1, replicates are divided by ","; IJC_SAMPLE_2 counts of inclusion junction in SAMPLE_2, replicates are divided by ","; SJC_SAMPLE_2：counts of skipping junction in SAMPLE_2; IncFormLen：Valid length of inclusion form; SkipFormLen: Valid length of skipping form; P-Value: Significancy in alternative splicing events between two samples; FDR: FDR value; IncLevel1: Inclusion level of samples in group SAMPLE_1 (replicates are divided by ","); IncLevel2: Inclusion level of samples in group SAMPLE_2 (replicates are divided by ","); IncLevelDifference：average(IncLevel1) – average(IncLevel2).

rMATS software analysis results directory:

[BMK_8_DEG_Analysis/BMK_1_A_vs_B/BMK_6_diff_AS_analysis](file:///C:\\Users\\27846\\Desktop\\%E8%AE%BA%E6%96%87\\%E9%9A%8B%E6%96%87%E9%9D%99\\BMK_8_DEG_Analysis\\BMK_1_A_vs_B\\BMK_6_diff_AS_analysis)

[BMK_8_DEG_Analysis/BMK_1_A_vs_C/BMK_6_diff_AS_analysis](file:///C:\\Users\\27846\\Desktop\\%E8%AE%BA%E6%96%87\\%E9%9A%8B%E6%96%87%E9%9D%99\\BMK_8_DEG_Analysis\\BMK_1_A_vs_C\\BMK_6_diff_AS_analysis)

[BMK_8_DEG_Analysis/BMK_1_A_vs_D/BMK_6_diff_AS_analysis](file:///C:\\Users\\27846\\Desktop\\%E8%AE%BA%E6%96%87\\%E9%9A%8B%E6%96%87%E9%9D%99\\BMK_8_DEG_Analysis\\BMK_1_A_vs_D\\BMK_6_diff_AS_analysis)

Statistics of differential alternative splicing events:

Table14 Statistics of differential alternative splicing events

| **DEG Set** | **A3SS** | **A5SS** | **MXE** | **RI** | **SE** |
| --- | --- | --- | --- | --- | --- |
| A_vs_B | 0 | 0 | 10 | 0 | 234 |
| A_vs_C | 0 | 0 | 15 | 0 | 295 |
| A_vs_D | 0 | 0 | 18 | 0 | 369 |

Note: DEG set: Name of DEG set; The rest columns: number of DEGs in corresponding alternative splicing events; A3SS: Alternative 3' splice junction; A5SS: Alternative 5' splice junction; MXE: Mutually exclusive exons; RI: Intron retention; SE: Exon skipping.

Statistics of differential alternative splicing events directory:

[BMK_8_DEG_Analysis](file:///C:\\Users\\27846\\Desktop\\%E8%AE%BA%E6%96%87\\%E9%9A%8B%E6%96%87%E9%9D%99\\BMK_8_DEG_Analysis)

**13 DEU Analysis**

Differential Exon Usage (DEU) analysis aims at revealing differentially expressed genes at exon level. For experiments with biological replicates, DEXSeq is employed in DEU analysis. DEXSeq[[29]](file:///C:\\Users\\27846\\Desktop\\%E8%AE%BA%E6%96%87\\%E9%9A%8B%E6%96%87%E9%9D%99\\index_en.html#ref29) identifies differentially expressed gene by use of negative binomial generalized linear models (GLM). Threshold for differential expression was set as FDR<0.01.

Outputs of DEU analysis are as follows.

Table15 Outputs of DEU analysis

| **geneID** | **exonID** | **log2(FC)** | **pvalue** | **FDR** |
| --- | --- | --- | --- | --- |
| gene-evm.model.tig00000005_pilon_pilon_pilon.45 | E002 | -0.092 | 4.91929043960203e-55 | 4.36521435975486e-51 |
| gene-evm.model.tig00000005_pilon_pilon_pilon.45 | E004 | 0.24 | 2.69254860106187e-63 | 3.5839168154434e-59 |
| gene-evm.model.tig00000005_pilon_pilon_pilon.48 | E003 | -0.38 | 5.08260237378139e-15 | 2.41614210343633e-12 |
| gene-evm.model.tig00000005_pilon_pilon_pilon.246 | E001 | 0.34 | 6.73760289789432e-09 | 1.23697742582651e-06 |
| gene-evm.model.tig00000005_pilon_pilon_pilon.246 | E002 | -0.12 | 1.42420595856433e-09 | 3.00903070023342e-07 |
| gene-evm.model.tig00000005_pilon_pilon_pilon.267 | E002 | 0.018 | 4.70645134783611e-05 | 0.0038 |
| gene-evm.model.tig00000005_pilon_pilon_pilon.312 | E002 | 0.12 | 0.00013 | 0.0086 |
| gene-evm.model.tig00000005_pilon_pilon_pilon.312 | E006 | -1.7 | 3.14251024204237e-14 | 1.24860843512552e-11 |
| gene-evm.model.tig00000005_pilon_pilon_pilon.432 | E001 | 0.019 | 1.16318209434947e-05 | 0.0011 |

Note: geneID: Gene ID;
exonID: Exon ID;
Log2(FC): log2(Fold change);
pvalue: Significancy of difference;
FDR: False discovery rate.

Demo figures of DEU were as follows:

Fig30 Demo result of DEU analysis

Note: (A) Fitted expression. The plot represents the expression estimates from a call to testForDEU.Shown in red is the exon that showed significant differential exon usage.(B) Transcripts. As in Figure A, but including the annotated transcript models.(C) Normalized counts. As in Figure A, with normalized count values of each exon in each of the samples. (D) Fitted splicing. The plot represents the estimated effects, as in Figure A, but after subtraction of overall changes in gene expression.

Outputs of DEU analysis directory:

[BMK_8_DEG_Analysis/BMK_1_A_vs_B/BMK_7_DEU_analysis](file:///C:\\Users\\27846\\Desktop\\%E8%AE%BA%E6%96%87\\%E9%9A%8B%E6%96%87%E9%9D%99\\BMK_8_DEG_Analysis\\BMK_1_A_vs_B\\BMK_7_DEU_analysis)

[BMK_8_DEG_Analysis/BMK_1_A_vs_C/BMK_7_DEU_analysis](file:///C:\\Users\\27846\\Desktop\\%E8%AE%BA%E6%96%87\\%E9%9A%8B%E6%96%87%E9%9D%99\\BMK_8_DEG_Analysis\\BMK_1_A_vs_C\\BMK_7_DEU_analysis)

[BMK_8_DEG_Analysis/BMK_1_A_vs_D/BMK_7_DEU_analysis](file:///C:\\Users\\27846\\Desktop\\%E8%AE%BA%E6%96%87\\%E9%9A%8B%E6%96%87%E9%9D%99\\BMK_8_DEG_Analysis\\BMK_1_A_vs_D\\BMK_7_DEU_analysis)

**14 Appendix**

**14.1 Appendix1: Software list**

Table16 Software list

| **Tools** | **Description** | **Linkages** |
| --- | --- | --- |
| HISAT2 | A spliced read mapper for RNA-Seq | http://ccb.jhu.edu/software/hisat2/index.shtml |
| StringTie | Transcript assembly for RNA-Seq | https://ccb.jhu.edu/software/stringtie/index.shtml |
| ASprofile | ASprofile is a suite of programs for extracting, quantifying and comparing alternative splicing (AS) events from RNA-seq data | http://ccb.jhu.edu/software/ASprofile/ |
| BLAST | Basic Local Alignment Search Tool | http://blast.ncbi.nlm.nih.gov/Blast.cgi |
| DESeq | An R package for RNA-Seq Differential Expression Analysis based on a model using the negative binomial distribution | http://www.bioconductor.org/packages/release/bioc/html/DESeq.html |
| EBSeq | An R package for RNA-Seq Differential Expression Analysis based on Bayesian approach | https://www.biostat.wisc.edu/~kendzior/EBSEQ/ |
| Cytoscape | An open source software platform for visualizing complex networks | http://www.cytoscape.org/ |
| topGO | An R package for gene ontology enrichment analysis | # |
| rMATs | MATS is a computational tool to detect differential alternative splicing events from RNA-Seq data. | http://rnaseq-mats.sourceforge.net/ |
| TFBStools | An R package for the analysis and manipulation of transcription factor binding sites. | http://www.bioconductor.org/packages/release/bioc/html/TFBSTools.html |

Note: # represents no links for software at the third column. The bioinformatic analysis softwares not given in the report are developed by us, and are not shown with the table.

**14.2 Appendix2: Database list**

Table17 Database table

| **Database** | **Description** | **Homepage** |
| --- | --- | --- |
| NR | non-redundant protein sequence database | ftp://ftp.ncbi.nih.gov/blast/db/ |
| Swiss-Prot | A manually annotated, non-redundant protein sequence database | http://www.uniprot.org/ |
| GO | Gene Ontology database | http://www.geneontology.org/ |
| COG | The database of Clusters of Orthologous Groups of proteins | http://www.ncbi.nlm.nih.gov/COG/ |
| KOG | The database of Clusters of Protein homology | http://www.ncbi.nlm.nih.gov/KOG/ |
| Pfam | The database of Homologous protein family | http://pfam.xfam.org/ |
| KEGG | The database of Kyoto Encyclopedia of Genes and Genomes | http://www.genome.jp/kegg/ |
| STRING | Search Tool for the Retrieval of Interacting Genes/Proteins | http://www.string-db.org/ |
| Ensembl | Database Sscrofa10.2 download from | http://asia.ensembl.org/index.html |
| Cosmic | COSMIC, is the world's largest and most comprehensive resource for exploring the impact of somatic mutations in human cancer. | https://cancer.sanger.ac.uk/cosmic |
| JASPAR | Database of transcription factor binding profiles | http://jaspar.genereg.net/ |

**14.3 Appendix3: Nucleic acid coding list**

Table18 Nucleic acid coding table

| **Nucleic Acid Code** | **Meaning** | **Mnemonic** |
| --- | --- | --- |
| A | A | Adenine |
| C | C | Cytosine |
| G | G | Guanine |
| T | T | Thymine |
| U | U | Uracil |
| R | A or G | puRine |
| Y | C, T or U | pYrimidines |
| K | G, T or U | bases which are Ketones |
| M | A or C | bases with aMino groups |
| S | C or G | Strong interaction |
| W | A, T or U | Weak interaction |
| B | not A (i.e. C, G, T or U) | Bcomes after A |
| D | not C (i.e. A, G, T or U) | Dcomes after C |
| H | not G (i.e., A, C, T or U) | Hcomes after G |
| V | neither T nor U (i.e. A, C or G) | Vcomes after U |
| N | A C G T U | Nucleic acid |

**14.4 Appendix4: Description on annotation databases**

Table19 Description on annotation databases

| **Database name** | **Database description** |
| --- | --- |
| NR database | Non-redundant protein database in NCBI, including Swissprot, PIR (Protein Information Resource), PRF (Protein Research Foundation), PDB (Protein Data Bank) protein database and CDS from GenBank and RefSeq |
| Swissprot database | A database maintained by EBI (European Bioinformatics Institute) containing a collated database of protein annotation information with relevant references and high credibility |
| COG database | A database for homologous classification of gene products. It is an early database for the identification of orthologous genes, which is obtained by comparing a large number of protein sequences of various organisms. |
| KOG database | For eukaryotes, homologous genes from different species are divided into different Ortholog clusters based on gene orthologous relationships and evolutionary relationships. Currently, KOG has 4852 classifications. Genes from the same Ortholog have the same function, so that functional annotations can be directly inherited to other members of the same KOG cluster. |
| Pfam database | The most comprehensive classification system for protein domain annotations. Proteins are composed of domains, and the protein sequences of each particular domain are somewhat conserved. Pfam divides the protein domain into different protein families, and establishes an HMM statistical model of the amino acid sequence of each family through alignment of protein sequences. |
| GO database | The internationally standardized gene function classification system provides a dynamically updated standard vocabulary to fully describe the functional properties of genes and gene products in organisms. There are three main categories of the database, namely molecular function, cellular component and biological process, each describing the molecular function that the gene product may perform, and the cellular environment and Participation in biological processes. The most basic concept in the GO database is Term, each entry has a Term name, such as "cell", "fibroblast growth factor receptor binding" or "signal transduction", with a unique number, like GO:nnnnnnn |
| KEGG database | A database that systematically analyzes the metabolic pathways of gene products in cells and the function of these gene products. It integrates data on genomics, chemical molecules, and biochemical systems, including PATHWAY, DRUG, DISEASE, GENES, and GENOME. Using this database helps to study the genes and their expressions as a whole network. |

Note: # represents no links for software at the third column. The bioinformatic analysis softwares not given in the report are developed by us, and are not shown with the table.

**14.5 Appendix5：High quality articles**

Biomarker has compiled the original texts and interpretations of high-scoring articles on related products, hoping to provide reference for your subject ideas. For details, please click the link to view：https://drive.weixin.qq.com/s?k=AOMAzQfDAAgJAon5xS

**14.6 Appendix6: Materials and methods**

**1 RNA extraction**

The plant total RNA was extracted using the RNAprep Pure Plant Kit (Tiangen, Beijing, China) according the instructions provided by the manufacturer.

The animal total RNA was extracted according to the instruction manual of the TRlzol Reagent (Life technologies, California, USA).

**2 Sample collection and preparation**

**2.1 RNA quantification and qualification**

RNA concentration and purity was measured using NanoDrop 2000(Thermo Fisher Scientific, Wilmington, DE). RNA integrity was assessed using the RNA Nano 6000 Assay Kit of the Agilent Bioanalyzer 2100 system (Agilent Technologies, CA, USA).

**2.2 Library preparation for Transcriptome sequencing**

A total amount of 1 μg RNA per sample was used as input material for the RNA sample preparations. Sequencing libraries were generated using NEBNext UltraTM RNA Library Prep Kit for Illumina (NEB, USA) following manufacturer’s recommendations and index codes were added to attribute sequences to each sample. Briefly, mRNA was purified from total RNA using poly-T oligo-attached magnetic beads. Fragmentation was carried out using divalent cations under elevated temperature in NEBNext First Strand Synthesis Reaction Buffer（5X）. First strand cDNA was synthesized using random hexamer primer and M-MuLV Reverse Transcriptase. Second strand cDNA synthesis was subsequently performed using DNA Polymerase I and RNase H . Remaining overhangs were converted into blunt ends via exonuclease/polymerase activities. After adenylation of 3’ ends of DNA fragments, NEBNext Adaptor with hairpin loop structure were ligated to prepare for hybridization. In order to select cDNA fragments of preferentially 240 bp in length, the library fragments were purified with AMPure XP system (Beckman Coulter, Beverly, USA). Then 3 μl USER Enzyme (NEB, USA) was used with size-selected, adaptor-ligated cDNA at 37°C for 15 min followed by 5 min at 95°C before PCR. Then PCR was performed with Phusion High-Fidelity DNA polymerase, Universal PCR primers and Index (X) Primer. At last, PCR products were purified (AMPure XP system) and library quality was assessed on the Agilent Bioanalyzer 2100 system.

**2.3 Clustering and sequencing**

The clustering of the index-coded samples was performed on a cBot Cluster Generation System using TruSeq PE Cluster Kit v4-cBot-HS (Illumia) according to the manufacturer’s instructions. After cluster generation, the library preparations were sequenced on an Illumina platform and paired-end reads were generated.

**3 Data analysis**

**3.1 Quality control**

Raw data (raw reads) of fastq format were firstly processed through in-house perl scripts. In this step, clean data(clean reads) were obtained by removing reads containing adapter, reads containing ploy-N and low quality reads from raw data. At the same time, Q20, Q30, GC-content and sequence duplication level of the clean data were calculated. All the downstream analyses were based on clean data with high quality.

**3.2 Comparative analysis**

The adaptor sequences and low-quality sequence reads were removed from the data sets. Raw sequences were transformed into clean reads after data processing. These clean reads were then mapped to the reference genome sequence. Only reads with a perfect match or one mismatch were further analyzed and annotated based on the reference genome. Hisat2 tools soft were used to map with reference genome.

**3.3 Gene functional annotation**

Gene function was annotated based on the following databases:Nr (NCBI non-redundant protein sequences)；Nt (NCBI non-redundant nucleotide sequences)；Pfam (Protein family)；KOG/COG (Clusters of Orthologous Groups of proteins)；Swiss-Prot (A manually annotated and reviewed protein sequence database)；KO (KEGG Ortholog database)；GO (Gene Ontology).

**3.4 Quantification of gene expression levels**

Quantification of gene expression levelsGene expression levels were estimated by fragments per kilobase of transcript per million fragments mapped. The formula is shown as follow:

*FPKM*=*cDNAFragmentsMappedFragments*(*Millions*)∗*TranscriptLength*(*kb*)

**3.5 Differential expression analysis**

For the samples with biological replicates:

Differential expression analysis of two conditions/groups was performed using the DESeq2. DESeq2 provide statistical routines for determining differential expression in digital gene expression data using a model based on the negative binomial distribution. The resulting P values were adjusted using the Benjamini and Hochberg’s approach for controlling the false discovery rate. Genes with an adjusted P-value < 0.01 found by DESeq2 were assigned as differentially expressed.

For the samples without biological replicates:

Differential expression analysis of two samples was performed using the edgeR.The FDR < 0.01 & Fold Change≥2 was set as the threshold for significantly differential expression.

**3.6 GO enrichment analysis**

Gene Ontology (GO) enrichment analysis of the differentially expressed genes (DEGs) was implemented by the GOseq R packages based Wallenius non-central hyper-geometric distribution（Young et al, 2010） ，which can adjust for gene length bias in DEGs.

**3.7 KEGG pathway enrichment analysis**

KEGG (Kanehisa et al., 2008) is a database resource for understanding high-level functions and utilities of the biological system, such as the cell, the organism and the ecosystem, from molecular-level information, especially large-scale molecular datasets generated by genome sequencing and other high-throughput experimental technologies (http://www.genome.jp/kegg/). We used KOBAS (Mao et al., 2005) software to test the statistical enrichment of differential expression genes in KEGG pathways.

**3.8 PPI (Protein Protein Interaction)**

The sequences of the DEGs was blast (blastx) to the genome of a related species (the protein protein interaction of which exists in the STRING database: http://string-db.org/) to get the predicted PPI of these DEGs. Then the PPI of these DEGs were visualized in Cytoscape (Shannon et al, 2003).

**References**

AltschulEwing B, Hillier L, Wendl S F, Madden T L, Schaffer A A, et al. (1997). Gapped BLAST and PSI-BLAST: a new generation of protein database search programs. Nucleic Acids Res. 25:3389-3402. (BLAST)

Anders S, Huber W. (2010).Differential expression analysis for sequence count data. Genome Biology,doi:10.1186/gb-2010-11-10-r106. (DESeq)

Finn R D, Tate J, Mistry J, et al. (2008). The Pfam protein families database. Nucleic Acids Res 36, D281-D288. (Pfam)

Gotz S, Garcia-Gomez J M, Terol J, et al. (2008).High-throughput functional annotation and data mining with the Blast2GO suite.Nucleic Acids Research 36, 3420-3435. (BLAST2go)

Mao X, Cai T, Olyarchuk J G, et al. (2005). Automated genome annotation and pathway identification using the KEGG Orthology (KO) as a controlled vocabulary.Bioinformatics 21, 3787-3793. (KOBAS)

Kanehisa M, Araki M, Goto S, et al. (2008). KEGG for linking genomes to life and the environment. Nucleic Acids research 36:D480-D484. (KEGG)

Love MI, Huber W and Anders S (2014). “Moderated estimation of fold change and dispersion for RNA-seq data with DESeq2.” Genome Biology, 15, pp. 550. doi: 10.1186/s13059-014-0550-8.(DESeq2)

Robinson M D, McCarthy D J, Smyth G K. (2010). edgeR: a Bioconductor package for differential expression analysis of digital gene expression data. Bioinformatics 26:139-140.(edgeR)

Young M D, Wakefield M J, Smyth G K, et al. (2010).Gene ontology analysis for RNA-seq: accounting for selection bias. Genome Biology, doi:10.1186/gb-2010-11-2-r14. (GOseq)

Shannon P, Markiel A, Ozier O, et al. (2003). Cytoscape: a software environment for integrated models of biomolecular interaction networks. Genome Res. 13, 2498-2504. (Cytoscape)

**References**

1. [Ewing B, Hillier L, Wendl MC, Green P. Base-calling of automated sequencer traces using phred. I. Accuracy assessment. Genome Research. 1998,8 (3): 175-185.](http://tour.biocloud.net/article/v1/into/articleDetail/9521921)
2. [Kim D, Langmead B, Salzberg S L. HISAT: a fast spliced aligner with low memory requirements[J]. Nature methods, 2015, 12(4): 357-360.](http://tour.biocloud.net/article/v1/into/articleDetail/25751142)
3. [Pertea M, Pertea G M, Antonescu C M, et al. StringTie enables improved reconstruction of a transcriptome from RNA-seq reads[J]. Nature biotechnology, 2015, 33(3): 290-295.](http://tour.biocloud.net/article/v1/into/articleDetail/25690850)
4. [Florea L, Song L, Salzberg S L. Thousands of exon skipping events differentiate among splicing patterns in sixteen human tissues. F1000Research, 2013, 2:188.](http://tour.biocloud.net/article/v1/into/articleDetail/24555089)
5. [Buchfink B, Xie C, Huson DH, "Fast and sensitive protein alignment using DIAMOND", Nature Methods 12, 59-60 (2015)](file:///C:\Users\27846\Desktop\%E8%AE%BA%E6%96%87\%E9%9A%8B%E6%96%87%E9%9D%99\index_en.html#a229)
6. [Deng YY, Li JQ, Wu SF, Zhu YP, et al. Integrated nr Database in Protein Annotation System and Its Localization. Computer Engineering. 2006, 32(5):71-74.](http://en.cnki.com.cn/Article_en/CJFDTOTAL-JSJC200605025.htm)
7. [Apweiler R, Bairoch A, Wu CH, et al. UniProt: the universal protein knowledgebase. Nucleic acids research. 2004, 32: D115-D119.](http://tour.biocloud.net/article/v1/into/articleDetail/27899622)
8. [Tatusov RL, Galperin MY, Natale D A. The COG database: a tool for genome scale analysis of protein functions and evolution. Nucleic Acids Research. 2000, 28(1):33-36.](http://tour.biocloud.net/article/v1/into/articleDetail/10592175)
9. [Koonin EV, Fedorova ND, Jackson JD, et al. A comprehensive evolutionary classification of proteins encoded in complete eukaryotic genomes. Genome biology. 2004, 5(2): R7.](http://tour.biocloud.net/article/v1/into/articleDetail/14759257)
10. [Kanehisa M, Goto S, Kawashima S, Okuno Y, et al. The KEGG resource for deciphering the genome. Nucleic Acids Research. 2004, 32:D277-D280.](http://tour.biocloud.net/article/v1/into/articleDetail/14681412)
11. [Jones P, Binns D, Chang H Y, et al. InterProScan 5: genome-scale protein function classification[J]. Bioinformatics, 2014, 30(9): 1236-1240.](http://tour.biocloud.net/article/v1/into/articleDetail/24451626)
12. [Ashburner M, Ball C A, Blake J A, et al. Gene ontology: tool for the unification of biology. Nature genetics. 2000, 25(1): 25-29.](http://tour.biocloud.net/article/v1/into/articleDetail/10802651)
13. [Eddy S R. Profile hidden Markov models. Bioinformatics, 1998, 14(9): 755-763.](http://tour.biocloud.net/article/v1/into/articleDetail/9918945)
14. [Finn RD, Bateman A, Clements J, et al. Pfam: the protein families database. Nucleic acids research. 2013: gkt1223.](http://tour.biocloud.net/article/v1/into/articleDetail/18039703)
15. [Trapnell C, Williams BA, Pertea G, Mortazavi A, et al. Transcript assembly and quantification by RNA Seq reveals unannotated transcripts and isoform switching during cell differentiation. Nature Biotechnology. 2010, 28(5):511-515.](http://tour.biocloud.net/article/v1/into/articleDetail/20436464)
16. [Djebali S, Davis CA, Merkel A, et al. Landscape of transcription in human cells. Nature. 2012, 489(7414): 101-108.](http://tour.biocloud.net/article/v1/into/articleDetail/22955620)
17. [Elowitz MB, Levine AJ, Siggia ED, Swain PS. Stochastic gene expression in a single cell. Science. 2002, 297:1183-1186.](http://tour.biocloud.net/article/v1/into/articleDetail/12183631)
18. [Kasper D. Hansen, Zhijin Wu, et al. Sequencing technology does not eliminate biological variability. Nature Biotechnology. 2011, 572-573.](http://tour.biocloud.net/article/v1/into/articleDetail/21747377)
19. [Robasky K, Lewis NE, Church GM. The role of replicates for error mitigation in next-generation sequencing. Nature Reviews Genetics. 2013.](http://tour.biocloud.net/article/v1/into/articleDetail/24322726)
20. [Insights into the correlation between Physiological changes in and seed development of tartary buckwheat (Fagopyrum tataricum Gaertn.). BMC Genomics. 2018 Aug 31;19(1):648.](http://tour.biocloud.net/article/v1/into/articleDetail/30170551)
21. [Love MI, Huber W and Anders S (2014). “Moderated estimation of fold change and dispersion for RNA-seq data with DESeq2.” Genome Biology, 15, pp. 550. doi: 10.1186/s13059-014-0550-8.](http://tour.biocloud.net/article/v1/into/articleDetail/25516281)
22. [Robinson MD, McCarthy DJ and Smyth GK (2010). edgeR: a Bioconductor package for differential expression analysis of digital gene expression data. Bioinformatics 26, 139-140](http://tour.biocloud.net/article/v1/into/articleDetail/19910308)
23. [Alexa A, Rahnenfuhrer J. topGO: enrichment analysis for gene ontology. R package version 2.8, 2010.](http://www.bioconductor.org/packages/2.11/bioc/html/topGO.html)
24. [Subramanian, A., Tamayo, P., Mootha, V. K., et al. Gene set enrichment analysis: a knowledge-based approach for interpreting genome-wide expression profiles. Proc. Natl. Acad. Sci. 2005, 102(43):15545–15550.](https://international.biocloud.net/zh/article/detail/16199517)
25. [Franceschini A, Szklarczyk D, Frankild S, et al. STRING v9. 1: protein-protein interaction networks, with increased coverage and integration. Nucleic acids research. 2013, 41: D808-D815.](http://tour.biocloud.net/article/v1/into/articleDetail/23203871)
26. [Altschul S F, Madden TL, Zhang J, et al. Gapped BLAST and PSI BLAST: A New Generation of Protein Database Search Programs. Nucleic Acids Research. 1997, 25(17): 3389-3402.](http://tour.biocloud.net/article/v1/into/articleDetail/9254694)
27. [Shannon P, Markiel A, Ozier O, et al. Cytoscape: a software environment for integrated models of biomolecular interaction networks. Genome research. 2003, 13(11): 2498-2504.](http://tour.biocloud.net/article/v1/into/articleDetail/14597658)
28. [Shen S., Park JW., Lu ZX., Lin L., Henry MD., Wu YN., Zhou Q., Xing Y.(2014) rMATS: Robust and Flexible Detection of Differential Alternative Splicing from Replicate RNA-Seq Data.PNAS, 111(51):E5593-601. doi: 10.1073/pnas.1419161111](http://tour.biocloud.net/article/v1/into/articleDetail/25480548)
29. [Anders S, Reyes A, Huber W. Detecting differential usage of exons from RNA-seq data. Genome research, 2012, 22(10): 2008-2017.](http://tour.biocloud.net/article/v1/into/articleDetail/22722343)
30. [Ge T, Boris L. TFBSTools: an R/bioconductor package for transcription factor binding site analysis:[J]. Bioinformatics, 2016, 32(10):1555-1556.](http://tour.biocloud.net/article/v1/into/articleDetail/26794315)
31. [Oriol Fornes, Jaime A Castro-Mondragon, Aziz Khan, et al. JASPAR 2020: update of the open-access database of transcription factor binding profiles [J]. Nucleic Acids Research, Volume 48, Issue D1, 08 January 2020, Pages D87-D92.](http://tour.biocloud.net/article/v1/into/articleDetail/31701148)
32. [Nicolle R, Radvanyi F, Elati M. COREGNET: reconstruction and integrated analysis of co-regulatory networks[J]. Bioinformatics, 2015:btv305.](http://tour.biocloud.net/article/v1/into/articleDetail/25979476)
33. [Ge H, Liu K, Juan T, et al. FusionMap: detecting fusion genes from next-generation sequencing data at base-pair resolution[J]. Bioinformatics, 2011, 27(14):1922-1928.](http://tour.biocloud.net/article/v1/into/articleDetail/21593131)

- [Abstract](file:///C:\Users\27846\Desktop\%E8%AE%BA%E6%96%87\%E9%9A%8B%E6%96%87%E9%9D%99\index_en.html#a8)
- [1 Experimental Procedure](file:///C:\Users\27846\Desktop\%E8%AE%BA%E6%96%87\%E9%9A%8B%E6%96%87%E9%9D%99\index_en.html#a9)
  - [1.1 RNA Quality Assessment](file:///C:\Users\27846\Desktop\%E8%AE%BA%E6%96%87\%E9%9A%8B%E6%96%87%E9%9D%99\index_en.html#a12)
  - [1.2 Library Construction](file:///C:\Users\27846\Desktop\%E8%AE%BA%E6%96%87\%E9%9A%8B%E6%96%87%E9%9D%99\index_en.html#a14)
  - [1.3 Library Quality Control](file:///C:\Users\27846\Desktop\%E8%AE%BA%E6%96%87\%E9%9A%8B%E6%96%87%E9%9D%99\index_en.html#a21)
  - [1.4 Sequencing](file:///C:\Users\27846\Desktop\%E8%AE%BA%E6%96%87\%E9%9A%8B%E6%96%87%E9%9D%99\index_en.html#a23)
- [2 Analysis Flow](file:///C:\Users\27846\Desktop\%E8%AE%BA%E6%96%87\%E9%9A%8B%E6%96%87%E9%9D%99\index_en.html#a25)
- [3 Data Quality Control](file:///C:\Users\27846\Desktop\%E8%AE%BA%E6%96%87\%E9%9A%8B%E6%96%87%E9%9D%99\index_en.html#a29)
  - [3.1 Sequencing Quality Control](file:///C:\Users\27846\Desktop\%E8%AE%BA%E6%96%87\%E9%9A%8B%E6%96%87%E9%9D%99\index_en.html#a33)
  - [3.2 Sequencing bases quality score](file:///C:\Users\27846\Desktop\%E8%AE%BA%E6%96%87\%E9%9A%8B%E6%96%87%E9%9D%99\index_en.html#a38)
  - [3.3 Nucleotide Distribution on Reads](file:///C:\Users\27846\Desktop\%E8%AE%BA%E6%96%87\%E9%9A%8B%E6%96%87%E9%9D%99\index_en.html#a48)
  - [3.4 Sequencing Data Statistics](file:///C:\Users\27846\Desktop\%E8%AE%BA%E6%96%87\%E9%9A%8B%E6%96%87%E9%9D%99\index_en.html#a52)
- [4 Data Alignment to Reference Genome](file:///C:\Users\27846\Desktop\%E8%AE%BA%E6%96%87\%E9%9A%8B%E6%96%87%E9%9D%99\index_en.html#a59)
  - [4.1 Mapping Statistics](file:///C:\Users\27846\Desktop\%E8%AE%BA%E6%96%87\%E9%9A%8B%E6%96%87%E9%9D%99\index_en.html#a65)
  - [4.2 Summary on Mapping](file:///C:\Users\27846\Desktop\%E8%AE%BA%E6%96%87\%E9%9A%8B%E6%96%87%E9%9D%99\index_en.html#a70)
- [5 Library Quality Control](file:///C:\Users\27846\Desktop\%E8%AE%BA%E6%96%87\%E9%9A%8B%E6%96%87%E9%9D%99\index_en.html#a76)
  - [5.1 mRNA Fragmentation Randomness Check](file:///C:\Users\27846\Desktop\%E8%AE%BA%E6%96%87\%E9%9A%8B%E6%96%87%E9%9D%99\index_en.html#a81)
  - [5.2 Length Distribution of Inserts](file:///C:\Users\27846\Desktop\%E8%AE%BA%E6%96%87\%E9%9A%8B%E6%96%87%E9%9D%99\index_en.html#a86)
  - [5.3 Saturation Test on RNA Sequencing Data](file:///C:\Users\27846\Desktop\%E8%AE%BA%E6%96%87\%E9%9A%8B%E6%96%87%E9%9D%99\index_en.html#a92)
- [6 Alternative Splicing Prediction](file:///C:\Users\27846\Desktop\%E8%AE%BA%E6%96%87\%E9%9A%8B%E6%96%87%E9%9D%99\index_en.html#a97)
  - [6.1 Statistics of Alternative Splicing Events](file:///C:\Users\27846\Desktop\%E8%AE%BA%E6%96%87\%E9%9A%8B%E6%96%87%E9%9D%99\index_en.html#a114)
  - [6.2 Alternative Splicing Pattern](file:///C:\Users\27846\Desktop\%E8%AE%BA%E6%96%87\%E9%9A%8B%E6%96%87%E9%9D%99\index_en.html#a118)
- [7 Gene Structure Optimization](file:///C:\Users\27846\Desktop\%E8%AE%BA%E6%96%87\%E9%9A%8B%E6%96%87%E9%9D%99\index_en.html#a121)
- [8 Novel Gene Analysis](file:///C:\Users\27846\Desktop\%E8%AE%BA%E6%96%87\%E9%9A%8B%E6%96%87%E9%9D%99\index_en.html#a125)
  - [8.1 Novel Gene Discovery](file:///C:\Users\27846\Desktop\%E8%AE%BA%E6%96%87\%E9%9A%8B%E6%96%87%E9%9D%99\index_en.html#a126)
  - [8.2 Functional Annotation of Novel Genes](file:///C:\Users\27846\Desktop\%E8%AE%BA%E6%96%87\%E9%9A%8B%E6%96%87%E9%9D%99\index_en.html#a131)
- [9 Gene Expression Quantification](file:///C:\Users\27846\Desktop\%E8%AE%BA%E6%96%87\%E9%9A%8B%E6%96%87%E9%9D%99\index_en.html#a136)
  - [9.1 Gene Expression Quantification](file:///C:\Users\27846\Desktop\%E8%AE%BA%E6%96%87\%E9%9A%8B%E6%96%87%E9%9D%99\index_en.html#a137)
  - [9.2 Distribution of Gene Expression](file:///C:\Users\27846\Desktop\%E8%AE%BA%E6%96%87\%E9%9A%8B%E6%96%87%E9%9D%99\index_en.html#a143)
  - [9.3 Correlation assessment of biological replicates](file:///C:\Users\27846\Desktop\%E8%AE%BA%E6%96%87\%E9%9A%8B%E6%96%87%E9%9D%99\index_en.html#a150)
- [10 Differential Expression Analysis](file:///C:\Users\27846\Desktop\%E8%AE%BA%E6%96%87\%E9%9A%8B%E6%96%87%E9%9D%99\index_en.html#a156)
- [11 Enrichment Analysis of DEGs](file:///C:\Users\27846\Desktop\%E8%AE%BA%E6%96%87\%E9%9A%8B%E6%96%87%E9%9D%99\index_en.html#a176)
  - [11.1 COG Classification on DEGs](file:///C:\Users\27846\Desktop\%E8%AE%BA%E6%96%87\%E9%9A%8B%E6%96%87%E9%9D%99\index_en.html#a179)
  - [11.2 GO Analysis on DEGs](file:///C:\Users\27846\Desktop\%E8%AE%BA%E6%96%87\%E9%9A%8B%E6%96%87%E9%9D%99\index_en.html#a184)
  - [11.3 GO Enrichment Analysis on DEGs](file:///C:\Users\27846\Desktop\%E8%AE%BA%E6%96%87\%E9%9A%8B%E6%96%87%E9%9D%99\index_en.html#a189)
  - [11.4 KEGG annotation of differentially expressed genes](file:///C:\Users\27846\Desktop\%E8%AE%BA%E6%96%87\%E9%9A%8B%E6%96%87%E9%9D%99\index_en.html#a193)
  - [11.5 KEGG Pathway enrichment analysis on DEGs](file:///C:\Users\27846\Desktop\%E8%AE%BA%E6%96%87\%E9%9A%8B%E6%96%87%E9%9D%99\index_en.html#a200)
  - [11.6 GSEA analysis](file:///C:\Users\27846\Desktop\%E8%AE%BA%E6%96%87\%E9%9A%8B%E6%96%87%E9%9D%99\index_en.html#a205)
  - [11.7 Protein-protein Interaction Network of DEGs](file:///C:\Users\27846\Desktop\%E8%AE%BA%E6%96%87\%E9%9A%8B%E6%96%87%E9%9D%99\index_en.html#a209)
- [12 Differential Alternative Splicing Analysis](file:///C:\Users\27846\Desktop\%E8%AE%BA%E6%96%87\%E9%9A%8B%E6%96%87%E9%9D%99\index_en.html#a214)
- [13 DEU Analysis](file:///C:\Users\27846\Desktop\%E8%AE%BA%E6%96%87\%E9%9A%8B%E6%96%87%E9%9D%99\index_en.html#a222)
- [14 Appendix](file:///C:\Users\27846\Desktop\%E8%AE%BA%E6%96%87\%E9%9A%8B%E6%96%87%E9%9D%99\index_en.html#a229)
  - [14.1 Appendix1: Software list](file:///C:\Users\27846\Desktop\%E8%AE%BA%E6%96%87\%E9%9A%8B%E6%96%87%E9%9D%99\index_en.html#a230)
  - [14.2 Appendix2: Database list](file:///C:\Users\27846\Desktop\%E8%AE%BA%E6%96%87\%E9%9A%8B%E6%96%87%E9%9D%99\index_en.html#a232)
  - [14.3 Appendix3: Nucleic acid coding list](file:///C:\Users\27846\Desktop\%E8%AE%BA%E6%96%87\%E9%9A%8B%E6%96%87%E9%9D%99\index_en.html#a234)
  - [14.4 Appendix4: Description on annotation databases](file:///C:\Users\27846\Desktop\%E8%AE%BA%E6%96%87\%E9%9A%8B%E6%96%87%E9%9D%99\index_en.html#a236)
  - [14.5 Appendix5：High quality articles](file:///C:\Users\27846\Desktop\%E8%AE%BA%E6%96%87\%E9%9A%8B%E6%96%87%E9%9D%99\index_en.html#a238)
  - [14.6 Appendix6: Materials and methods](file:///C:\Users\27846\Desktop\%E8%AE%BA%E6%96%87\%E9%9A%8B%E6%96%87%E9%9D%99\index_en.html#a240)
- [References](file:///C:\Users\27846\Desktop\%E8%AE%BA%E6%96%87\%E9%9A%8B%E6%96%87%E9%9D%99\index_en.html#js-reference)

Biomarker Technologies (BMK) GmbH, All Copyrights Reserved.

Address：Technologiepark Münster, Johann-Krane-Weg 42, 48149 Münster, Germany

- E-mail:[tech@bmkgene.com](mailto:tech@bmkgene.com)
- Linkedin:[Biomarker (BMKGENE) Europe](https://www.linkedin.com/company/bmkgene-eu)
- [About us](https://www.bmkgene.com)
