# Supplementary material for: Cloning and expression of the pkg1 gene from the GH55 family of the mycoparasite Pestalotiopsis kenyana PG52
Source: Front Microbiol. 2025 Oct 23;16:1665330. doi: 10.3389/fmicb.2025.1665330 (PMC12590764; doi:10.3389/fmicb.2025.1665330)
Supplement: Supplementary file 2 [file Supplementary_file_2.docx]

Appendix I

Tab. I-1 The FPKM value of glucanase genes in PG52 strains

| **Gene ID** | **0h** | | | **24h** | | | **48h** | | | **72h** | | |
| --- | --- | --- | --- | --- | --- | --- | --- | --- | --- | --- | --- | --- |
| *pkg1* | 7.501 | 7.156 | 6.399 | 6.778 | 8.306 | 9.214 | 4.567 | 8.122 | 4.366 | 3.798 | 4.150 | 3.469 |
| *pkg2* | 0.482 | 0.402 | 0.203 | 0.167 | 0.274 | 0.317 | 0.167 | 0.081 | 0.071 | 0.113 | 0.205 | 0.148 |
| *pkg3* | 13.609 | 13.438 | 12.837 | 17.233 | 17.023 | 17.968 | 20.113 | 22.201 | 15.798 | 14.606 | 13.639 | 14.534 |
| *pkg4* | 0.278 | 0.346 | 0.307 | 0.082 | 0.066 | 0.269 | 0.140 | 0.155 | 0.194 | 0.117 | 0.299 | 0.123 |
| *pkg5* | 2.153 | 2.584 | 2.238 | 1.029 | 1.782 | 1.520 | 1.049 | 0.901 | 0.808 | 0.981 | 0.607 | 0.663 |
| *pkg6* | 2.354 | 2.822 | 2.588 | 0.485 | 0.355 | 0.430 | 0.169 | 0.425 | 0.282 | 0.074 | 0.145 | 0.104 |
| *pkg7* | 0.037 | 0.018 | 0.000 | 0.000 | 0.029 | 0.000 | 0.015 | 0.033 | 0.059 | 0.000 | 0.000 | 0.000 |
| *pkg8* | 0.000 | 0.120 | 0.071 | 0.422 | 0.489 | 0.456 | 0.000 | 0.109 | 0.000 | 0.000 | 0.185 | 0.213 |
| *pkg9* | 0.000 | 0.000 | 0.173 | 0.093 | 0.061 | 0.000 | 0.000 | 0.093 | 0.164 | 0.061 | 0.245 | 0.351 |
| *pkg10* | 23.353 | 19.665 | 21.231 | 28.131 | 25.683 | 29.201 | 24.430 | 24.102 | 23.051 | 80.839 | 79.018 | 71.141 |
| *pkg11* | 59.449 | 57.758 | 58.631 | 62.617 | 65.894 | 55.195 | 65.212 | 76.484 | 57.623 | 94.910 | 102.514 | 95.451 |
| *pkg12* | 768.884 | 696.449 | 670.055 | 415.438 | 377.455 | 455.664 | 343.915 | 390.702 | 284.915 | 305.666 | 333.980 | 313.050 |
| *pkg13* | 376.934 | 361.738 | 353.210 | 370.798 | 391.182 | 462.494 | 496.426 | 525.618 | 439.872 | 774.765 | 897.246 | 830.163 |
| *pkg14* | 367.156 | 372.541 | 370.477 | 589.605 | 640.623 | 556.706 | 352.953 | 312.892 | 458.874 | 252.566 | 236.536 | 257.138 |
| *pkg15* | 65.032 | 67.947 | 65.535 | 55.829 | 65.155 | 48.460 | 50.946 | 55.284 | 53.781 | 91.794 | 91.381 | 96.021 |
| *pkg16* | 102.316 | 98.191 | 98.057 | 110.852 | 118.061 | 85.019 | 93.231 | 81.491 | 99.183 | 52.323 | 48.894 | 48.656 |
| *pkg17* | 0.057 | 0.000 | 0.145 | 0.000 | 0.000 | 0.000 | 0.346 | 0.247 | 0.109 | 0.448 | 0.305 | 0.403 |
| *pkg18* | 0.029 | 0.000 | 0.000 | 0.000 | 0.000 | 0.032 | 0.030 | 0.000 | 0.036 | 0.016 | 0.103 | 0.107 |

Continued Table I-1 FPKM Values of Glucanase Genes in Two Strains of Bacteria

| **Gene ID** | **0h** | | | **24h** | | | **48h** | | | **72h** | | |
| --- | --- | --- | --- | --- | --- | --- | --- | --- | --- | --- | --- | --- |
| *pkg19* | 0.000 | 0.050 | 0.069 | 0.228 | 0.203 | 0.000 | 0.390 | 0.210 | 0.141 | 0.102 | 0.000 | 0.000 |
| *pkg20* | 9.819 | 10.251 | 9.436 | 8.029 | 8.872 | 9.576 | 4.242 | 5.044 | 5.047 | 3.723 | 3.387 | 3.247 |
| *pkg21* | 0.000 | 0.000 | 0.079 | 0.076 | 0.000 | 0.000 | 0.035 | 0.000 | 0.000 | 0.000 | 0.000 | 0.000 |
| *pkg22* | 3.096 | 4.330 | 3.029 | 3.948 | 6.607 | 1.418 | 3.849 | 1.742 | 4.220 | 3.864 | 3.027 | 4.632 |
| *pkg23* | 2.231 | 1.473 | 1.662 | 1.109 | 0.763 | 1.582 | 1.135 | 1.627 | 0.854 | 1.943 | 2.394 | 2.389 |
| *pkg24* | 3.450 | 3.366 | 3.125 | 1.159 | 1.419 | 1.267 | 1.369 | 1.232 | 0.917 | 0.948 | 1.498 | 0.803 |
| *pkg25* | 24.070 | 19.912 | 23.598 | 6.561 | 8.482 | 9.165 | 11.992 | 16.303 | 14.143 | 22.971 | 28.930 | 21.638 |
| *pkg26* | 1.045 | 1.153 | 1.269 | 0.595 | 0.640 | 0.555 | 0.270 | 0.490 | 0.320 | 0.049 | 0.062 | 0.124 |
| *pkg27* | 1.914 | 1.509 | 1.954 | 3.989 | 2.438 | 2.287 | 1.846 | 2.063 | 2.065 | 1.243 | 1.397 | 0.908 |
| *pkg28* | 109.520 | 99.644 | 103.731 | 83.532 | 82.733 | 80.792 | 81.387 | 93.587 | 69.693 | 49.790 | 45.153 | 46.104 |
| *pkg29* | 0.041 | 0.000 | 0.037 | 0.000 | 0.017 | 0.060 | 0.000 | 0.000 | 0.000 | 0.000 | 0.000 | 0.000 |
| *pkg30* | 27.211 | 23.458 | 24.183 | 4.111 | 4.716 | 6.554 | 1.921 | 3.009 | 0.933 | 2.700 | 3.970 | 3.156 |
| *pkg31* | 0.000 | 0.072 | 0.000 | 0.037 | 0.018 | 0.000 | 0.000 | 0.000 | 0.060 | 0.000 | 0.000 | 0.000 |
| *pkg32* | 0.728 | 1.124 | 0.748 | 0.784 | 0.864 | 1.065 | 0.163 | 0.291 | 0.439 | 0.099 | 0.160 | 0.080 |
| *pkg33* | 3.455 | 3.691 | 3.502 | 2.676 | 1.926 | 4.750 | 3.242 | 4.131 | 1.837 | 2.048 | 2.615 | 2.306 |
| *pkg34* | 45.338 | 65.414 | 57.121 | 183.355 | 172.223 | 117.701 | 52.006 | 44.453 | 70.367 | 87.525 | 64.311 | 89.216 |
| *pkg35* | 0.327 | 0.254 | 0.086 | 0.000 | 0.183 | 0.000 | 0.220 | 0.206 | 0.165 | 0.063 | 0.328 | 0.161 |
| *pkg36* | 0.000 | 0.000 | 0.092 | 0.000 | 0.000 | 0.000 | 0.000 | 0.000 | 0.000 | 0.000 | 0.000 | 0.000 |
| *pkg37* | 44.687 | 46.116 | 47.695 | 69.056 | 72.589 | 67.709 | 69.985 | 54.802 | 72.191 | 17.539 | 15.612 | 15.580 |
| *pkg38* | 394.291 | 371.428 | 376.952 | 329.842 | 342.947 | 319.210 | 289.412 | 294.342 | 277.663 | 317.801 | 332.321 | 298.200 |
| *pkg39* | 0.119 | 0.029 | 0.059 | 0.077 | 0.000 | 0.000 | 0.015 | 0.069 | 0.016 | 0.055 | 0.117 | 0.061 |
| *pkg40* | 372.348 | 398.584 | 389.282 | 329.917 | 345.662 | 372.789 | 276.783 | 338.686 | 264.808 | 399.784 | 471.418 | 411.540 |
| *pkg41* | 251.721 | 230.609 | 243.821 | 284.625 | 280.407 | 304.894 | 120.308 | 116.018 | 151.776 | 85.740 | 82.379 | 86.626 |
| *pkg42* | 30.965 | 25.824 | 28.492 | 36.836 | 30.372 | 32.776 | 45.204 | 57.173 | 33.303 | 44.555 | 48.022 | 42.008 |
| *pkg43* | 31.291 | 30.596 | 29.947 | 37.691 | 37.617 | 40.529 | 16.931 | 20.323 | 16.171 | 11.996 | 11.852 | 10.162 |
| *pkg44* | 2.479 | 1.529 | 2.310 | 1.712 | 1.886 | 2.083 | 2.145 | 2.177 | 1.094 | 1.824 | 1.712 | 1.593 |
| *pkg45* | 23.292 | 19.510 | 21.928 | 3.581 | 2.894 | 3.387 | 4.435 | 6.334 | 3.563 | 16.431 | 16.914 | 14.165 |

Continued Table I-1 FPKM Values of Glucanase Genes in Two Strains of Bacteria

| **Gene ID** | **0h** | | | **24h** | | | **48h** | | | **72h** | | |
| --- | --- | --- | --- | --- | --- | --- | --- | --- | --- | --- | --- | --- |
| *pkg46* | 1398.81 | 1410.52 | 1377.81 | 1565.17 | 1670.61 | 1240.44 | 1788.86 | 1647.63 | 2213.91 | 2066.39 | 1785.82 | 2077.20 |
| *pkg47* | 22.909 | 24.962 | 23.106 | 24.274 | 26.573 | 9.445 | 26.964 | 19.453 | 28.903 | 7.646 | 4.728 | 6.125 |

Appendix II

>*pkg1*

MGSSHHHHHHSSGLVPRGSHMASMTGGQQMGRGSEFTDGQQQQQPAATSSFWYANLDHTGPYRGYAPGLGANSSTYSVYKAVAPGASAVEIQAAINDDDNGGARHSQWLASQPRVVYIPPGTYEINQTIYFNTDTILMGDATDPPVFKAVADGFADDQTLVSGQDPGTGEQGELSFAVGLKNIVLDTTAIDGGTSFTALWWGVAQGAQLQNVKITMASSVNGQGHSGIRLGRGSTLGLSDVRIERGQNGIWHNGHQQASYKSIYFYENTVGMLIDGGYTISLLAPTFETVGTAVSCTGGYPWIAIVDGKSINSGVTFNTTSYPSLLIENLVKDTDSDIAVVADVGTVLAAATHVDQFSYANTVGRDPIYGAVSSSSGRPAALAPGGYYPVVPAPNYAAYPVSDFVNVKDPSQNGGHTVLGDASGDDAAALNAALAYAAAQHKIAYFPFGKYRVDDTLVIPAGSRVVGEAWATISGSGAGGNFLDAANPQPVVRVGSAGDVGTVAQIQDMRFTVADVLPGAIVLQFEAAGLQPGDVGLWNSLVTVGGTRGSLDGESDECADAADQCRAAFLGIHLAPTSSAYIENVWNWVADHATEGEEGGSHIAGKGGVLVQATRGTWLYALGSEHWWLYQLNLFGASNVAVSLLQSETNYDQGSNAEQVAPAPWTPDAAGWGDPDFSWCEGISNKTEAALCSMGLANYIQGGENIYTYASASWAFFSGPGYQACEGNCQDVVHWIAETPDNLQAFGLCSKDTYAGLRLANGTDIVTEDGFTGSWGGDVGRYTP

>*ITS* mRNA

TCGTAACAAGGTCTCCGTAGGTGAACCTGCGGAGGGATCATTACAAGTGACCCCGGTCTAACCACCGGGATGTTCATAACCCTTTGTTGTCCGACTCTGTTGCCTCCGGGGCGACCCTGCCTTCGGGCGGGGGCTCCGGGTGGACACTTCAAACTCTTGCGTAACTTTGCAGTCTGAGTAAACTTAATTAATAAATTAAAACTTTTAACAACGGATCTCTTGGTTCTGGCATCGATGAAGAACGCAGCGAAATGCGATAAGTAATGTGAATTGCAGAATTCAGTGAATCATCGAATCTTTGAACGCACATTGCGCCCCCTGGTATTCCGGGGGGCATGCCTGTTCGAGCGTCATTTCACCACTCAAGCCTCGCTTGGTATTGGGCAACGCGGTCCGCCGCGTGCCTCAAATCGACCGGCTGGGTCTTCTGTCCCCTAAGCGTTGTGGAAACTATTCGCTAAAGGGTGTTCGGGAGGCTACGCCGTAAAACAACCCCATTTCTAAGGTTGACCTCGGATCAGGTAGGGATACCCGCTGAACTTAAGCATATCA

>*Actin* mRNA

ATGGAAGAGGAGGTCGCTGCCCTCGTTATCGACAATGGTTCGGGTATGTGCAAGGCCGGTTTCGCCGGTGATGATGCTCCCCGAGCTGTTTTCCCCTCCATTGTCGGTCGCCCCCGTCACCATGGTATCATGATTGGTATGGGTCAGAAGGACTCGTATGTCGGTGATGAGGCACAGTCGAAGCGTGGTATCCTGACGCTGCGCTACCCCATTGAGCACGGTGTTGTCACCAACTGGGACGACATGGAGAAGATCTGGCACCACACCTTCTACAACGAGCTGCGTGTTGCCCCTGAGGAGCACCCCGTCCTGCTCACTGAGGCTCCCATCAACCCAAAGTCCAACCGTGAGAAGATGACCCAGATTGTCTTTGAGACCTTCAACGCCCCGGCTTTCTACGTCTCCATCCAGGCCGTCCTGTCTCTGTACGCCTCCGGTCGTACCACCGGTATCGTGCTCGACTCCGGTGATGGTGTCACCCACGTTGTCCCCATCTACGAGGGTTTCGCCCTGCCCCACGCCATTGCCCGTGTGGATATGGCCGGTCGTGACTTGACCGACTACCTCATGAAGATTCTGGCCGAGCGTGGTTACTCCTTCTCCACCACTGCCGAGCGTGAAATCGTTCGTGACATCAAGGAGAAGCTCTGCTACGTCGCCCTCGACTTTGAGCAGGAGATCCAGACTGCTGCCCAGAGCTCCAGCTTGGAGAAGTCCTACGAGCTTCCCGACGGTCAGGTCATCACCATTGGCAACGAGCGTTTCCGTGCTCCTGAGGCTCTCTTCCAGCCCTCTGTTCTGGGTCTTGAGAGCGGTGGTATCCACGTTACCACTTACAACTCCGTCATGAAGTGCGATGTCGATGTCCGTAAGGATCTGTACGGAAACATTGTCATGTCTGGTGGTACCACCATGTACCCCGGTCTGTCCGACCGTATGCAGAAGGAGATCACTGCCCTGGCCCCGTCTTCCATGAAGGTCAAGATCATTGCTCCTCCTGAGCGCAAGTACTCCGTCTGGATCGGTGGTTCCATTCTCGCTTCCCTGTCGACCTTCCAGCAAATGTGGATCTCGAAGCAGGAGTACGACGAGAGTGGTCCTTCCATTGTCCACCGCAAGTGCTTCTAA
